# Long non-coding RNAs PGM5-AS1 upregulates Decorin (DCN) to inhibit cervical cancer progression by sponging miR-4284

**DOI:** 10.1080/21655979.2022.2062088

**Published:** 2022-04-14

**Authors:** Huimin Wang, Dan Wang, Qiong Wei, Chun Li, Chunyan Li, Jing Yang

**Affiliations:** a Department of Obstetrics and Gynecology, Renmin Hospital of Wuhan University, Wuhan, Hubei, China; b Department of Obstetrics and Gynecology, Wuhan Third Hospital, Tongren Hospital of Wuhan University, Wuhan, Hubei, China

**Keywords:** lncRNA PGM5-AS1, cervical cancer, miR-4284, DCN

## Abstract

Long non-coding RNAs (lncRNAs) have been widely studied and play crucial roles in cervical cancer (CC) progression. Here, we investigated the function and mechanism of lncRNA PGM5-AS1 action in CC cells. Using real-time quantitative polymerase chain reaction or western blotting, PGM5-AS1 and decorin (DCN) were downregulated in CC tissues and cells, whereas miR-4284 was upregulated. Luciferase assay, RNA pull-down assay, and western blotting showed that PGM5-AS1 could sponge miR-4284 to upregulate DCN expression in CC cells. Additionally, cell functional experiments showed that PGM5-AS1 overexpression led to decreased proliferation, migration, and invasion of CC cells. However, the inhibitory effect of PGM5-AS1 overexpression on CC cells was partly relieved by DCN knockdown because of the targeting interaction between PGM5-AS1, miR-4284, and DCN. In summary, this study identified that PGM5-AS1 negatively regulates CC cell malignancy by targeting miR-4284/DCN.

## GRAPHICAL ABSTRACT

**Figure:**
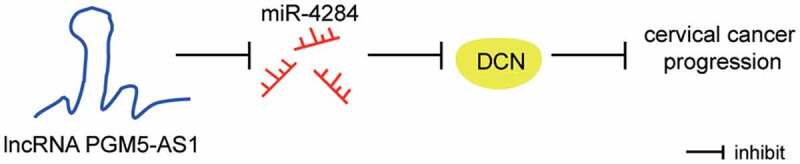

## Highlights

- PGM5-AS1 is an anti-cancer lncRNA in CC.
- PGM5-AS1 can sponge miR-4284 to regulate DCN.
- DCN knockdown partly revises the effect of PGM5-AS1 overexpression on CC cells.

## Introduction

Cervical cancer (CC) is among the deadliest diseases worldwide owing to its high morbidity and mortality [1,2]. Many factors, including human papillomavirus (HPV) infection, changes in gene expression, smoking, and sexual hygiene can cause CC [3–6]. Despite numerous efforts to prevent and treat CC [7,8], such as HPV screening, vaccination, and radical hysterectomy, the curative and survival rates of CC patients are still poor, especially in developing countries [2]. Therefore, a complete understanding of the mechanism of CC is beneficial for its diagnosis and treatment.

Long non-coding RNAs (lncRNAs) have been discovered to act as sponges of microRNAs (miRNAs) to regulate miRNA expression by competitive endogenous RNAs (ceRNA) mechanisms, thereby affecting tumor development [9,10]. For example, lncRNA CAR10 exerts a promoting function for CC by sponging miR-125b-5p to enhance PDPK1 expression [11]. The lncRNA CTS targets the miR-505/ZEB2 axis to aggravate metastasis and epithelial-to-mesenchymal transition in CC [12]. The lncRNA PTAR-binding miR-101 can inhibit apoptosis and promote proliferation in CC [13]. PGM5-AS1 is an lncRNA and has been found to act differently in different cancers. In prostate cancer, PGM5-AS1 plays an anti-cancer role; its overexpression suppresses proliferation and facilitates apoptosis [14]. In osteosarcoma, PGM5-AS1 induces epithelial-mesenchymal transition and invasion of osteosarcoma cells, thereby playing an oncogenic role [15]. We used the GEPIA database to analyze the expression of PGM5-AS1 in cancers and found that PGM5-AS1 was downregulated in many cancers, especially CC. However, a literature review did not reveal the function of PGM5-AS1 in CC. Therefore, we decided to investigate the effects of PGM5-AS1 on CC. MiRNAs are small non-coding RNAs that are regulated by lncRNAs, thereby regulating cancer progression [16–18]. MiR-106b is reportedly regulated by lncRNA PTENP1, thereby contributing to CC progression by accelerating cell proliferation and epithelial-mesenchymal transition [19]. MiR-125b-5p, which is suppressed by the lncRNA CAR10, attenuates CC cell malignancy [11]. Here, we performed bioinformatics analysis and identified that miR-4284 may be regulated by PGM5-AS4 in CC. After reviewing the literature, miR-4284 was found to act as an oncogenic miRNA in gastric cancer [20], non-small cell lung cancer [21], and glioblastoma [22]. Nevertheless, the interaction between miR-4284 and lncRNA PGM5-AS1 has not yet been identified in CC.

Bioinformatic analysis is often used to identify the key genes involved in human diseases [23,24]. For example, the GEO database was first used to identify 250 differentially expressed genes (DEGs) and the enrichment of gene ontology (GO) and KEGG pathways further identified seven key genes associated with atherosclerosis [25]. Here, we used an mRNA microarray GSE9750 from the GEO database to screen 393 downregulated genes in CC samples. Finally, we found that DCN targeted by miR-4284 was the key gene in CC by GO enrichment. Decorin (DCN), a member of the small leucine-rich proteoglycan family, is an extracellular matrix protein that regulates multiple cellular processes, such as angiogenesis [26], autophagy [27], and tumor growth [28]. In lung adenocarcinoma, DCN is downregulated in tissues and suppresses the proliferation of lung adenocarcinoma cells [29]. Hu et al. found that DCN overexpression suppressed tumorigenesis and metastasis in inflammatory breast cancer [30]. However, the action mechanism of DCN in CC remains unknown.

In this study, we aimed to identify the functions and action mechanisms of PGM5-AS1 in CC. Bioinformatic analysis suggested that the miR-4284/DCN axis might be related to the PGM5-AS1 mechanism in CC. Therefore, performing cell functional experiments to identify the function and mechanism of PGM5-AS1 in CC would be useful as a potential therapeutic target for CC.

## Materials and methods

### Bioinformatics analysis

GEPIA (http://gepia.cancer-pku.cn/index.html) is an online tool that displays the expression of PGM5-AS1 in human cancer types. GSE9750 [31] from GEO DataSets was applied to identify the downregulated genes in CC samples after setting the adj.P < 0.01 and logFC≤-2. Another online tool, Metascape (http://metascape.org/gp/index.html#/main/step1), was used to perform GO enrichment of downregulated genes in CC samples. UALCAN (http://ualcan.path.uab.edu/index.html), which stores data from TCGA, was used to further identify the expression of key genes in CC. Finally, the miRNAs binding to PGM5-AS1 and the key gene DCN were predicted using miRDB and miRWalk, respectively.

### Clinical samples collection and cell culture

CC samples and adjacent normal samples were collected from 29 patients diagnosed with CC at Wuhan Third Hospital. The tissue samples were stored in liquid nitrogen. All patients with CC provided written informed consent, and the study was approved by the Ethics Committee of Wuhan Third Hospital (approval number: KY2022-013).

All cell lines, including the CC cell lines HeLa (BNCC340384) and CaSki (BNCC338223), as well as the human immortalized epidermal cell line HaCaT (BNCC340423), were purchased from BeNa Culture Collection (Beijing, China). HaCaT cells were cultured in DMEM-H, whereas CC cells were cultured in RPMI-1640. Medium containing 10% FBS was added, and all cells were incubated in a cell incubator at 37°C and 5% CO_2_.

### Real-time quantitative polymerase chain reaction (qRT-PCR)

TRIzol reagent (Invitrogen, USA) was used to isolate total RNA from tissues and cells, and either a cDNA synthesis kit (Roche, USA) or a one-step miRNA RT kit (Haigene, China) was used to synthesize 1 μg of RNA to cDNA. qRT-PCR was performed to measure the expression of lncRNA, miRNA and mRNA using Real-Time PCR Master Mix (Genema, China). The relative expression levels of lncRNA, miRNA, and mRNA were calculated using the 2^−∆∆Ct^ method [32] and normalized to GAPDH or U6 as an internal reference. Supplementary Table 1 lists all primer sequences. Agarose gel electrophoresis was used to analyze the primers of qRT-PCR.

### Subcellular fractionation assay

The location of PGM5-AS1 in CC cells was identified using a subcellular fractionation assay with the PARIS kit (Ambion, USA). Briefly, CC cells were collected and washed with PBS, and 500 μL of cell fractionation buffer was added to resuspend the cells. After cell dissolution on ice for 15 min, the cells were centrifuged and the cytoplasm in the supernatant was separated from the cell nucleus in the sediment. qRT-PCR was used to detect the PGM5-AS1 expression in the cytoplasm and nucleus [33].

### Cell transfection

PGM5-AS1 overexpression vectors (pcDNA-PGM5-AS1) and empty pcDNA3.1, as negative controls (NC), were purchased from RiboBio (China). Three siRNAs targeting DCN (si-DCN-1, si-DCN-2, and si-DNC-3), miR-4284 mimic, and NC were purchased from RiboBio (China). The siRNAs sequences the targeting DCN were as follows: si-DCN-1 forward 5’-GAUUCUUGUCAACAAUAAA-3’ and reverse 5’-UUUAUUGUUGACAAGAAUC-3’; si-DCN-2 forward 5’-CAUUGAUUCUUGUCAACAA-3’ and reverse 5’-UUGUUGACAAGAAUCAAUG-3’, and si-DCN-3 forward 5’-CCAGAUGAUUGUCAU AGAA-3’ and reverse 5’-UUCUAUGACAAUC AUCUGG-3’. When the cell confluence was >70%, pcDNA-PGM5-AS1, si-DCN, miR-4284 mimic, and NC were transfected into HeLa and CaSki cells using Lipofectamine 3000 (Invitrogen). qRT-PCR was performed to determine the transfection efficiency after 48 h.

### EDU cell proliferation assay

This assay was performed to assess the change in cell proliferation after transfection using the EDU DNA Cell Proliferation Kit (RiboBio, China) [34]. Briefly, CC cells were seeded in 96-well plates and transfected for 48 h. EDU (50 μM/well) was added to the cells for 2 h and then fixed with 4% paraformaldehyde. After fixing, the cells were stained with Apollo dye solution, and nucleic acids in the cells were stained with DAPI. Finally, the stained cells were imaged using a fluorescence microscope.

### Wound healing assay

The assay was performed as previously described [35]. The transfected CC cells (3 × 10^6^ cells/well) were cultured in 6-well plates and incubated until the cell confluence reached 100%. The cell monolayers were then scratched with 200 μL pipette tips. After the floating cells were removed using PBS, the cells were incubated for 24 h in serum-free culture medium. Images of the wound were captured at 0 and 24 h, and the migration rate was measured using the following formula: (W_0h_−W_24 h_)/W_0 h_ × 100%, where W represents the wound width.

### Transwell assay

Transwell chambers (Corning, USA) were used for this assay [35]. After transfection, CC cells (5 × 104 cells/well) were inoculated into the upper chambers that were paved with 8% Matrigel. The cells in the upper chambers were incubated in medium without FBS, whereas medium with 10% FBS was added to the lower chamber. After incubation for 24 h, the cells that invaded the lower surface of the membrane were fixed with 4% paraformaldehyde for 15 min and stained with crystal violet for 15 min. The invading cells were photographed at 250× magnification using a light microscope.

### RNA pull-down assay

This assay was performed using the Pierce RNA 3’ End Desthiobiotinylation Kit (Thermo Fisher Scientific), and biotinylated vectors (bio-NC, bio-miR-4709-3p, bio-miR-5701, bio-miR-3686, bio-miR-450b-5p, bio-miR-587, bio-miR-4695-3p, bio-miR-4284, bio-miR-6743-5p, bio-miR-4688, bio-miR-8059, and bio-miR-627-3p) were purchased from RiboBio (China). Briefly, HeLa cells (2 × 10^4^ cells/well) were plated in 24-well plates and transfected with 50 nM biotinylated vectors using Lipofectamine 3000 (Invitrogen). After 48 h of transfection, the transfected cells were lysed with lysis buffer and incubated with streptavidin magnetic beads (Life Technologies, USA) for 3 h. Finally, after washing the beads with PBS, RNA interacting with miRNA was extracted to identify DCN enrichment using qRT-PCR [19].

### Luciferase assay

Wild-type (WT) PGM5-AS1/DCN with a binding site and mutant-type (MUT) PGM5-AS1/DCN without binding sites were constructed by Tsingke Biotechnology Co., Ltd. (China), and inserted into pGL3 luciferase reporter vectors (Promega, USA). The abovementioned vectors were co-transfected with a miR-4284 mimic or mimic-NC into CC cells. The Dual-Luciferase Reporter Assay System was purchased from Promega (USA) to detect luciferase activities after 48 h cell co-transfection [14].

### Western blotting

Total protein isolation from CC cell lines was performed using RIPA lysis buffer (Invitrogen), and the protein concentration was measured using the BCA method. Then, 20 μg of total protein was separated using 10% SDS-PAGE and transferred to a PVDF membrane. After blocking, the membranes were incubated at 4°C with anti-GAPDH (1:1000, ab9485, Abcam, USA) and anti-DCN (1:1000, ab181456, Abcam, USA) overnight. Next, the membrane was incubated with fluorescent rabbit antibody (1:3000, 926–32,211, LI-COR Biosciences, Germany) for 3 h at 22°C. The protein blots were visualized using an Odyssey 3.2 infrared imaging system (LI-COR Biosciences, Germany) [36].

### Statistical analysis

All data from three independent experiments were analyzed using GraphPad Prism version 8 and are shown as the mean ± standard deviation. The comparison between the two groups was analyzed by paired t-test, whereas the comparison among more than two groups was analyzed using analysis of variance. Differences were considered statistically significant if the P-value was less than 0.05.

## Results

Using bioinformatics analysis, we hypothesized that PGM5-AS1 plays a key role in CC. Therefore, this study aimed to investigate the role and molecular mechanism of PGM5-AS1 in CC progression. Through a series of cell function experiments, we showed that PGM5-AS1 located in the cytoplasm could suppress CC cell malignancy. Additionally, we found that PGM5-AS1 could sponge miR-4284 to regulate DCN. Overall, our study revealed that PGM5-AS1 acts as an anti-cancer lncRNA in CC by sponging the miR-4284/DCN axis.

### PGM5-AS1 located in cytoplasm was downregulated in CC

**Figure 1.**
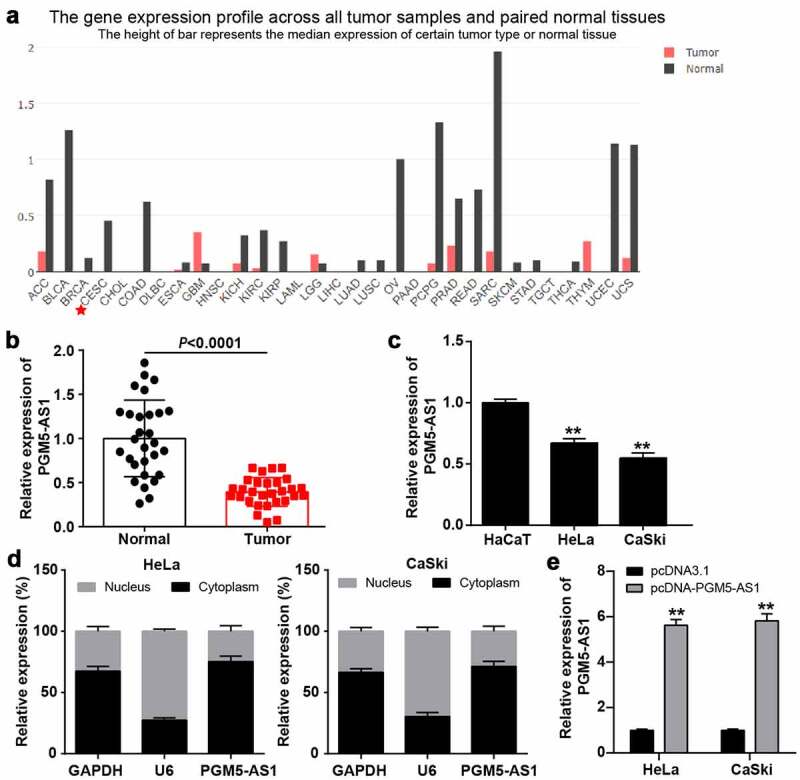
PGM5-AS1 located in cytoplasm was downregulated in CC. (a) GEPIA displayed the expression of PGM5-AS1 in human cancers. CESC, cervical squamous cell carcinoma and endocervical adenocarcinoma. (b) qRT-PCR detected the expression of PGM5-AS1 in clinical CC tissues and adjacent normal tissues. (c) qRT-PCR detected the expression of PGM5-AS1 in CC cell lines (HeLa and CaSki) and human immortalized epidermal cell line HaCaT. **P < 0.001 vs. HaCaT. (d) Subcellular fractionation assay identified the location of PGM5-AS1 in CC cells. (e) qRT-PCR identified the transfection efficiency of pcDNA-PGM5-AS1 in CC cells. **P < 0.001 vs. pcDNA3.1.

GEPIA analysis revealed that PGM5-AS1 was downregulated in most cancer types, especially in cervical squamous cell carcinoma and endocervical adenocarcinoma (CESC, Figure 1(a)). qRT-PCR was performed to verify the PGM5-AS1 expression in clinical CC and adjacent normal tissues, while the PGM5-AS1 expression was reduced by ~50% in CC tissues (Figure 1(b)). Similarly, PGM5-AS1 was downregulated in CC cell lines (HeLa and CaSki) compared to the human immortalized epidermal cell line HaCaT (Figure 1(c)). Subcellular fractionation assay revealed that PGM5-AS1 was mainly located in the cytoplasm of CC cells (Figure 1(d)). After transfecting pcDNA-PGM5-AS1 into CC cells, it was found that PGM5-AS1 expression was elevated by nearly 6-fold in the pcDNA-PGM5-AS1 group compared to that in the pcDNA3.1 group (Figure 1(e)).

### PGM5-AS1 overexpression suppressed CC cell malignancy

**Figure 2.**
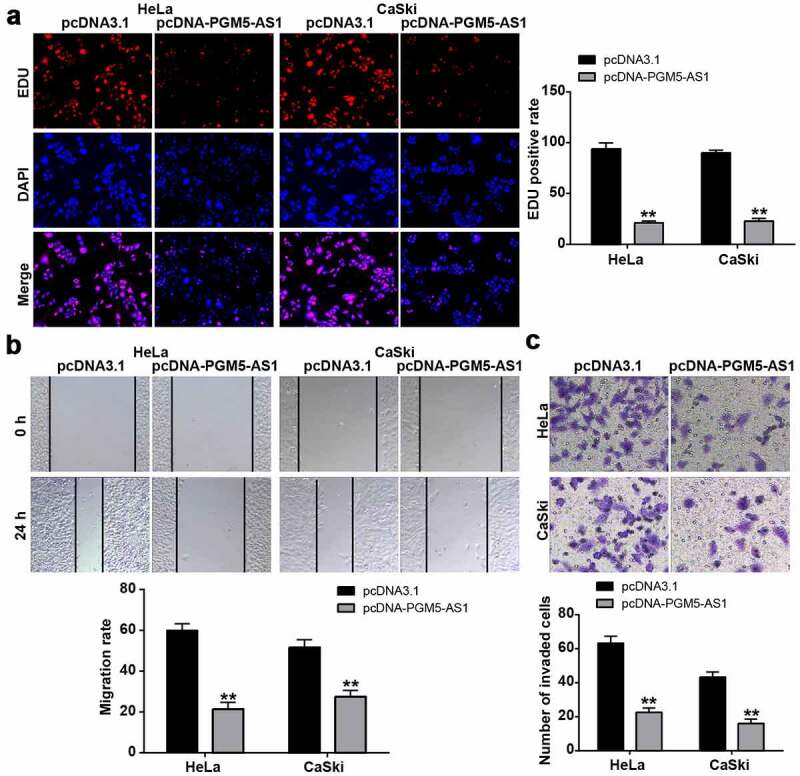
PGM5-AS1 overexpression suppressed CC cell malignancy. (a) EDU assay identified the change of cell proliferation in CC cells after transfecting with pcDNA-PGM5-AS1 vectors. (b) Wound healing assay measured the change of migration rate in CC cells after transfecting with pcDNA-PGM5-AS1 vectors. (c) Transwell assay verified the change of the number of invaded cells in CC cells after transfecting with pcDNA-PGM5-AS1 vectors. **P < 0.001 vs. pcDNA3.1.

The EDU assay was used to reflect the change in cell proliferation in CC cells after transfection with pcDNA-PGM5-AS1 vectors. The results indicated that the EDU-positive rate in the pcDNA-PGM5-AS1 group decreased by more than 70% (Figure 2(a)), suggesting that PGM5-AS1 inhibited cell proliferation. Cell migration ability was examined using a wound healing assay, which showed that PGM5-AS1 overexpression significantly reduced the migration rate of CC cells (P < 0.001, Figure 2(b)). Additionally, the results of the transwell assay revealed that the number of invaded cells in the pcDNA-PGM5-AS1 group was much lower than that in the pcDNA3.1 group (Figure 2(c)).

### miR-4284/DCN axis might be the downstream of PGM5-AS1 in CC

**Figure 3.**
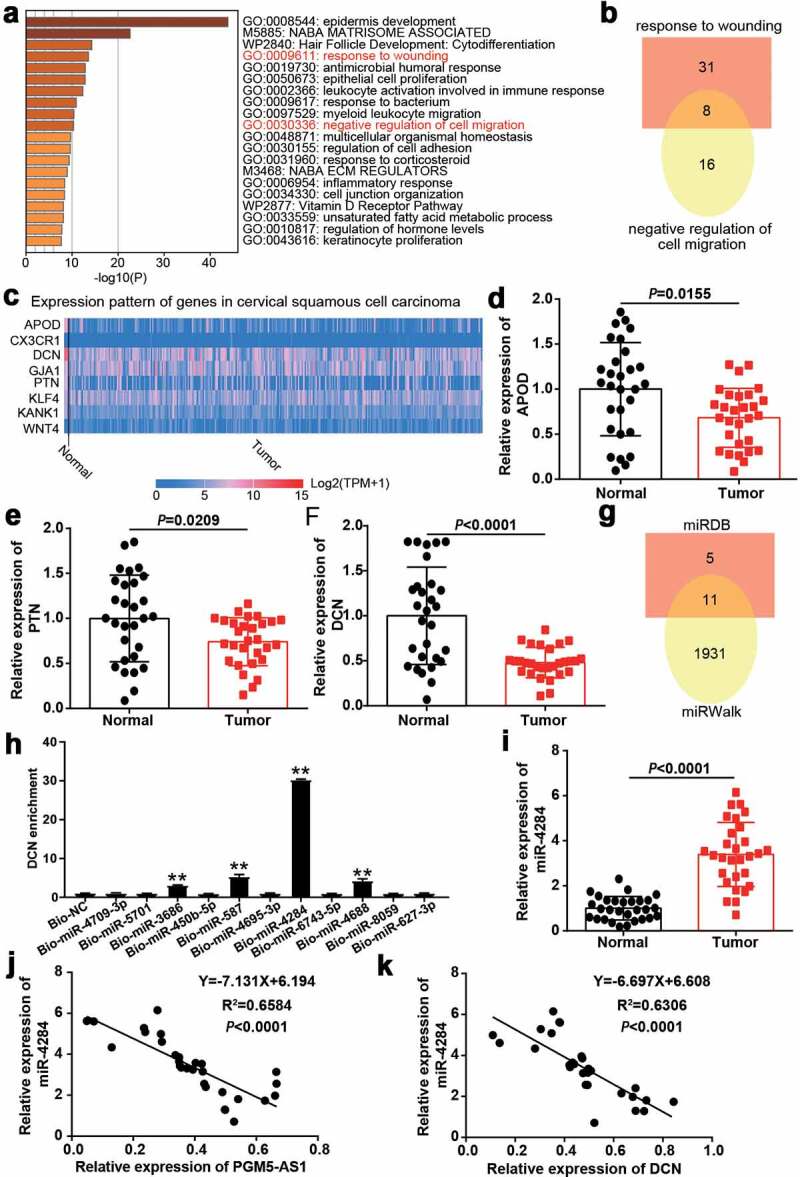
miR-4284/DCN axis might be the downstream of PGM5-AS1 in CC. (a) GO enrichment of 393 downregulated genes in CC was analyzed by Metascape. (b) Eight genes associated with wounding and cell migration were screened out. (c) The expression of eight genes in cervical squamous cell carcinoma was analyzed according to TCGA database including 3 normal samples and 305 tumor samples. Red, high expression in samples. Blue, low expression in samples. (d-f) qRT-PCR identified the expression of APOD, PTN and DCN in clinical CC tissue samples and adjacent normal samples. (g) A total of 11 miRNAs were overlapped from miRDB and miRWalk. miRDB was applied to predict the miRNAs binding to PGM5-AS1. miRWalk was applied to predict the miRNAs binding to PTN. (h) RNA pull-down assay identified the DNC enrichment in eleven miRNAs groups. Bio, biotinylated. NC, negative control. **P < 0.001 vs. Bio-NC. (i) qRT-PCR identified the expression of miR-4284 in clinical CC tissue samples and adjacent normal samples. (j-k) Pearson correlation analysis identified the negative correlation between miR-4284 expression and PGM5-AS1/DCN expression in clinical CC tissues.

A total of 393 downregulated genes in CC samples were screened from GSE9750 with the criteria of adj.P < 0.01 and logFC≤-2. The downregulated genes uploaded to Metascape for GO enrichment identified the wounding response and negative regulation of cell migration associated with cancer progression as key biological processes (Figure 3(a)). After overlapping the genes related to the abovementioned biological processes, a total of eight genes were screened (Figure 3(b)). The expressions of these eight genes in cervical squamous cell carcinoma were analyzed according to the TCGA database with three normal samples and 305 CC samples. The results showed that APOD, DCN, and PTN were downregulated in tumor samples compared with normal samples (Figure 3(c)). After performing qRT-PCR, we identified DCN as our gene of interest because it was significantly downregulated (P < 0.0001) in our clinical CC tissue samples compared to that in APOD (P = 0.0155) and PTN (P = 0.0209, Figure 3(d-f)). Next, miRDB was applied to predict miRNA binding to PGM5-AS1 (Supplementary table 2), whereas miRWalk (Supplementary table 3) was used to predict miRNA binding to DCN. As shown in Figure 3(g), 11 miRNAs (miR-4709-3p, miR-5701, miR-3686, miR-450b-5p, miR-587, miR-4695-3p, miR-4284, miR-6743-5p, miR-4688, miR-8059 and miR-627-3p) were identified as common miRNAs in the miRDB and miRWalk results. Using the RNA pull-down assay, DCN was found to be mainly enriched in the bio-miR-4284 group, indicating that miR-4284 could bind to DCN (Figure 3(h)). Therefore, miR-4284 was identified as the miRNA of interest. In clinical samples, miR-4284 was found to be overexpressed in CC samples (Figure 3(i)), and miR-4284 expression was negatively correlated with PGM5-AS1 or DCN expression in our clinical CC tissues (Figure 3(j,k)).

### PGM5-AS1 upregulated DCN expression by sponging miR-4284 in CC cells

**Figure 4.**
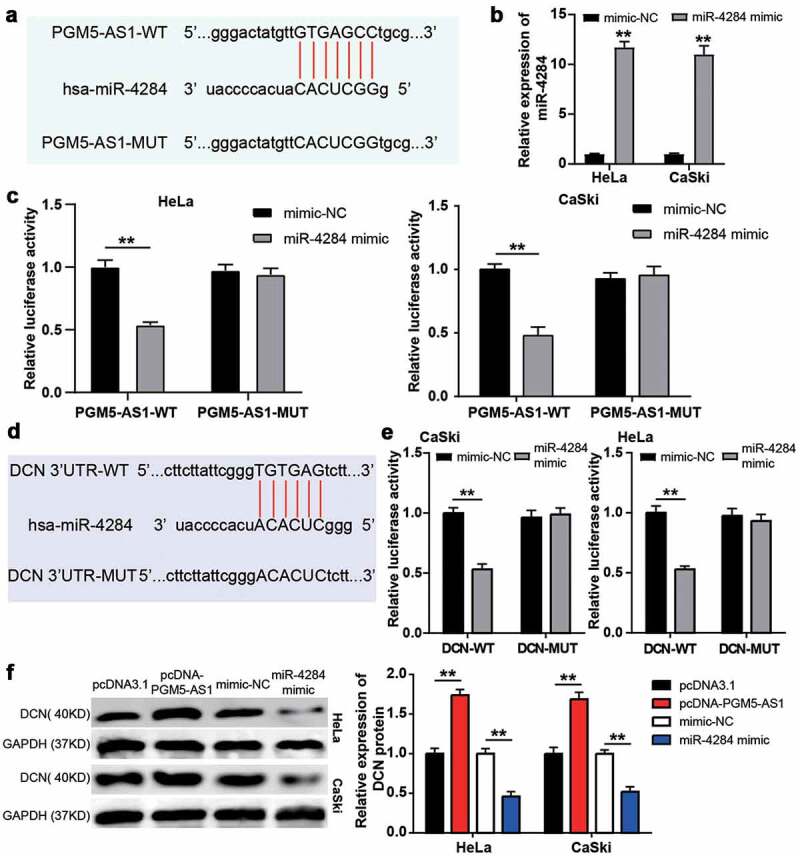
PGM5-AS1 upregulated DCN expression by sponging miR-4284 in CC cells. (a) The binding site between PGM5-AS1 and miR-4284. (b) qRT-PCR identified the transfection efficiency of miR-4284 mimic in CC cells. mimic, miR-4284 mimic. **P < 0.001 vs. mimic-NC. (c) The luciferase assay measured the luciferase activity in transfected CC cells. WT, wild type. MUT, mutant. mimic, miR-4284 mimic. **P < 0.001. (d) The binding site between DCN and miR-4284. (e) The luciferase assay measured the luciferase activity in transfected CC cells. WT, wild type. MUT, mutant. mimic, miR-4284 mimic. **P < 0.001. (f) Western blotting detected the expression of DCN protein in transfected CC cells. mimic, miR-4284 mimic. **P < 0.001.

The PGM5-AS1 and miR-4284 binding sites are shown in Figure 4(a). A luciferase assay was performed after successful transfection of the miR-4284 mimic into CC cell lines (HeLa and CaSki, Figure 4(b)); it was found that the miR-4284 mimic induced decreased luciferase activity of the PGM5-AS1-WT group, while the luciferase activity of the PGM5-AS1-MUT group was not affected by the miR-4284 mimic (Figure 4(c)). These data suggest that miR-4284 binds to PGM5-AS1. Next, miRWalk was used to predict the binding site between DCN and miR-4284 (Figure 4(d)). Based on the miRWalk results, we designed DCN-MUT and DCN-WT vectors to transfect HeLa and CaSki cells, respectively. The results showed that the luciferase activity in the co-transfection of miR-4284 mimic+DCN-WT group was reduced, but it did not affect whether the cells were transfected with DCN-MUT vectors (Figure 4(e)). These results confirmed the presence of binding sites between DCN and miR-627-3p. Finally, the western blotting assay proved that PGM5-AS1 overexpression upregulated the expression of the DCN protein, whereas the miR-4284 mimic downregulated the expression of the DCN protein (Figure 4(f)).

### DCN knockdown improved the passive influence of PGM5-AS1 overexpression on CC cells

**Figure 5.**
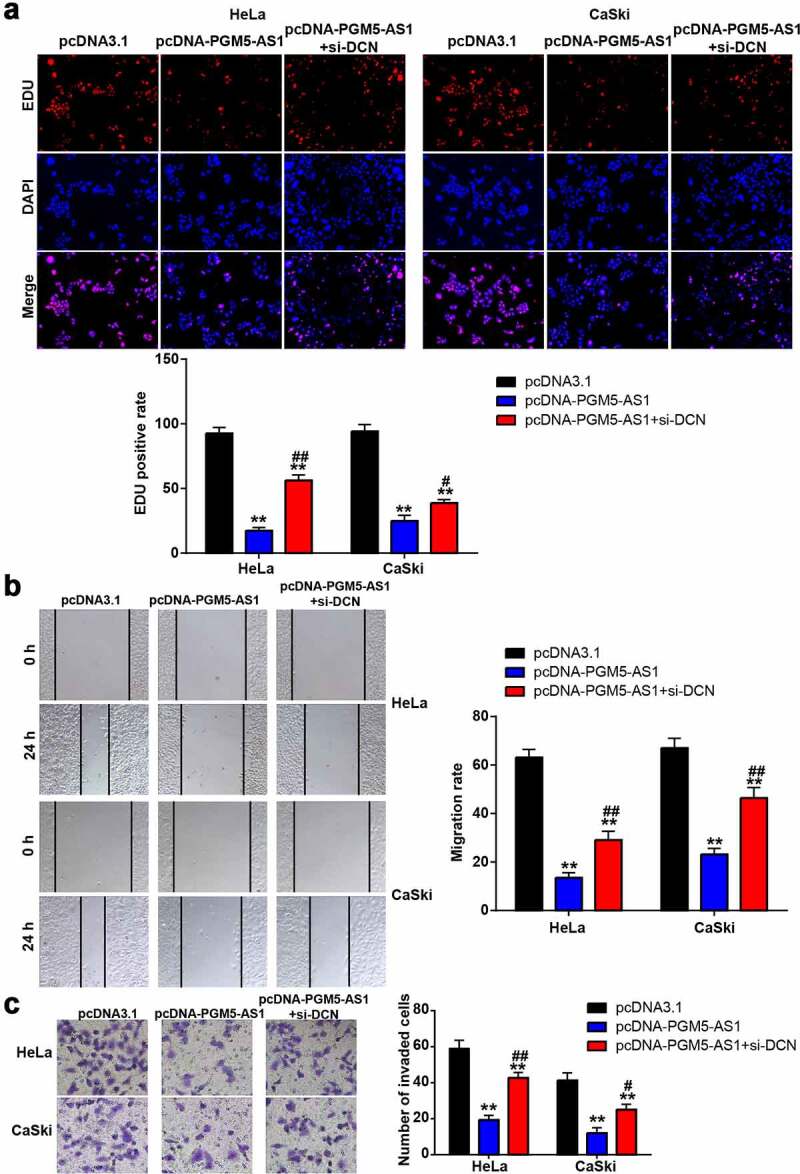
DCN knockdown improved the negative effect of PGM5-AS1 overexpression on CC cells. (a) EDU assay identified the change of cell proliferation in transfected CC cells. (b) Wound healing assay measured the change of migration rate in transfected CC cells. (c) Transwell assay verified the change of the number of invaded cells in transfected CC cells. **P < 0.001 vs. pcDNA3.1. #P < 0.05, ##P < 0.001 vs. pcDNA-PGM5-AS1.

To verify whether DCN, downstream of PGM5-AS1, affects the regulatory effect of PGM5-AS1 on CC cells, we designed three siRNAs targeting DCN (si-DCN-1, si-DCN-2, and si-DCN-3). After checking the transfection efficiency by qRT-PCR, it was found that si-DCN-1 had the highest transfection efficiency in HeLa and CaSki cells (Supplementary Figure S1); therefore, si-DCN-1 was used for subsequent cell function experiments. The EDU assay showed that the decrease in the EDU-positive rate induced by PGM5-AS1 overexpression was elevated by co-transfecting pcDNA-PGM5-AS1 and si-DCN (Figure 5(a)). Similar to cell proliferation, the cell migration rate in the pcDNA-PGM5-AS1+ si-DCN group was increased compared to the pcDNA-PGM5-AS1 group (Figure 5(b)). Transwell assays confirmed that co-transfection of pcDNA-PGM5-AS1 and si-DCN increased the number of invading cells compared to pcDNA-PGM5-AS1 (Figure 5(c)).

## Discussion

LncRNAs have been reported to participate in CC progression by targeting miRNAs to enhance mRNAs expression [37–39]. Here, we found that PGM5-AS1 was underexpressed in CC, and its upregulation suppressed the proliferation, migration, and invasion of CC cells. Additionally, our data showed that PGM5-AS1 could target miR-4284 to enhance DCN expression in CC cells, thereby suggesting that PGM5-AS1 acts as an anti-tumor lncRNA in CC by targeting the miR-4284/DCN axis.

GEPIA is a useful tool for screening key regulators in cancer and has been widely used in cancer studies [40–42]. In this study, PGM5-AS1 with low expression was screened as a key lncRNA in CC using GEPIA. After reviewing the literature, we found that PGM5-AS1 plays different roles in various cancers. For example, PGM5-AS1 is downregulated in prostate cancer and its overexpression impairs tumor growth in prostate cancer cells [14]. However, Liu et al. confirmed that PGM5-AS1 could enhance epithelial-mesenchymal transition and invasion in osteosarcoma cells, thereby playing a positive role in osteosarcoma [15]. In CC, we proved for the first time that PGM5-AS1 showed low expression and was an anti-tumor lncRNA in CC by inhibiting proliferation, migration, and invasion, thus enriching the function of PGM5-AS1 in human cancers.

An increasing number of studies have revealed that the regulatory network involving the ceRNA mechanism participates in the progression of multiple cancers [43]. For example, lncRNA CDC6 acts as a ceRNA to target CDC6 by sponging miR-215, thereby promoting breast cancer progression [17]. LINC01133 was found to be a ceRNA that targets the miR-106a-3p/APC axis to inhibit gastric cancer progression [44]. Previous studies have shown that PGM5-AS1 targets miR-484 in colorectal cancer [45] and miR-584 in prostate cancer [14]. In contrast to previous studies, our study confirmed that PGM5-AS1 could target miR-4284 to upregulate DCN expression, thereby inhibiting the proliferation, migration, and invasion of CC cells.

GSE9750, an mRNA microarray, was used to identify key CC genes [46–48]. In previous studies, researchers used more than three microarrays from the GEO database, including GSE9750, to screen key genes in CC [47–49]. When performing different bioinformatic strategies in our study, we only used GSE9750, along with metascape for GO enrichment, and the TCGA database to identify that DCN was the key gene in CC; this result was different from those of previous studies. Although the antitumor functions of DCN in lung adenocarcinoma [29] and breast cancer [30] have been identified, the DCN action mechanism in CC has not yet been explored. Together with bioinformatic analysis and cell function experiments, we confirmed that DCN is a key gene in CC progression. Further, DCN was shown to be downstream of PGM5-AS1, indicating that PGM5-AS1 overexpression could upregulate DCN expression by sponging miR-4284. Moreover, silencing DCN effectively relieved the inhibitory effect of PGM5-AS1 overexpression on CC cells.

Our study demonstrated the function of the PGM5-AS1/miR-4284/DCN axis in CC; however, there were still some limitations in our study. First, we only performed cell function experiments and the effect of the PGM5-AS1/miR-4284/DCN axis on CC, both clinically and in vivo, needs to be further explored. Second, the downstream signaling pathway involving DCN was deeply explored in this study, and needs to be identified in the future. Next, this study used only one mRNA microarray from the GEO database; more microarrays involving CC from the GEO database should be used to verify other key genes in the future. At the same time, epidermis development was the first result after performing GO enrichment in this study, which is needed to explore the effect of CC progression and might provide some new ideas in the future. Additionally, the luciferase assay results indicated that the interaction sequences between PGM5-AS1 and miR-4284and between miR-4284and DCN were the same, which may present a stable ternary complex to determine CC cell fate. This will be investigated in detail in our next study.

## Conclusion

In summary, this study is the first to show that PGM5-AS1 attenuates CC progression by regulating cell proliferation, migration, and invasion. Moreover, we showed that PGM5-AS1, mainly located in cell cytoplasm, competitively binds to miR-4284 to upregulate DCN in CC cells. Hence, our findings enrich the understanding of the function and mechanism of PGM5-AS1 in human cancers and provide a potential therapeutic target for CC.

## Supplementary Material

Supplemental Material

## Data Availability

All data and materials are available from the corresponding author if request is reasonable.
