# Supplementary material for: Long non-coding RNAs PGM5-AS1 upregulates Decorin (DCN) to inhibit cervical cancer progression by sponging miR-4284
Source: Bioengineered. 2022 Apr 14;13(4):9872–84. doi: 10.1080/21655979.2022.2062088 (PMC9161867; doi:10.1080/21655979.2022.2062088)
Supplement: Supplemental Material [file KBIE_A_2062088_SM1689.zip › supplementary/downloadFromZipFile.pdf]

武汉市第三医院伦理委员会审查批件

批件号：武三医伦 KY2022-013

项目名称	探究宫颈癌发展的分子机制		
项目来源	武汉市第三医院		
项目类型	药品☐ 器械☐ 科研项目☑ 医疗技术☐ 其他☐		
承担科室	妇产科	主要研究者	王会敏
评审资料	见附件送审清单		
审查类别	初始审查	审查方式	简易审查
审查时间	2022 年 3 月 11 日		
年度/定期跟踪审查频率（自试验批准日起）	3 个月☐ 6 个月☐ 12 个月☑ 无☐		

审查意见：

根据卫生部《涉及人的生物医学研究伦理审查办法(2016)》、国家药监局《药物临床试验质量管理规范(2020) 》、《医疗器械临床试验质量管理规范(2016) 》、WMA《赫尔辛基宣言》 和 CIOMS《人体生物医学研究国际道德指南》的伦理原则，经本伦理委员会审查，同意按所批准的临床研究方案、知情同意书等开展本项研究。

注意事项：

1. 本伦理批件自批准之日期起有效期一年；逾期未实施的本批件自行作废。
2. 研究应遵循 GCP 原则和伦理委员会批准的方案开展临床研究,保护受试者的健康与权益。
3. 研究过程中若变更主要研究者，对临床研究方案、知情同意书、招募材料等的任何修改，请申请人提交修正案审查。
4. 自同意研究日起，按照跟踪审查频率递交年度/定期跟踪审查报告，请在跟踪审查到期前 1 个月递交。
5. 发生严重不良事件，请申请人及时提交严重不良事件报告。
6. 重大违背或偏离方案应及时提交违背/偏离方案报告表。
7. 申请人暂停或提前终止临床研究，请及时提交暂停/终止研究报告。
8. 完成研究，请申请人提交研究完成报告。

武汉市第三医院伦理委员会（盖章）

主任委员/副主任委员签名：[signature]

2022 年 3 月 14 日

声明：武汉市第三医院伦理委员会的职责、人员组成、操作程序及记录遵循中华人民共和国食品药品监督管理局颁布的药物临床试验质量管理规范（GCP）和 ICH-GCP 的伦理审查原则，并遵守中国的相关法律及法规。

地址：武汉市武昌区彭刘杨路 241 号 邮编：430060 电话：027-68894978

扫描全能王 创建
