# Supplementary material for: Long non-coding RNAs PGM5-AS1 upregulates Decorin (DCN) to inhibit cervical cancer progression by sponging miR-4284
Source: Bioengineered. 2022 Apr 14;13(4):9872–84. doi: 10.1080/21655979.2022.2062088 (PMC9161867; doi:10.1080/21655979.2022.2062088)
Supplement: Supplemental Material [file KBIE_A_2062088_SM1689.zip › supplementary/Supplementary Table 1.docx]

Supplementary Table 1 The sequences of primers used in this study.

| **Primer** | **Sequences** |
| --- | --- |
| **PGM5-AS1** | Forward: 5′-GACTATGTTGTGAGCCTGCG-3′ |
|  | Reverse: 5′-AAAAGGGGAGGGGCAATACA-3′ |
| **DCN** | Forward: 5′-TGTTCTGATTTGGGTTGTCTACCT-3′ |
|  | Reverse: 5′-GGACCGGGTTGCTGAAAAGA-3′ |
| **miR-4284** | Forward: 5'-GCCGAGGGGCTCACATCACCCCAT-3' |
|  | Reverse: 5'-CTCAACTGGTGTCGTGGA-3' |
| **GAPDH** | Forward: 5'-GGGAAACTGTGGCGTGAT-3' |
|  | Reverse: 5'-GAGTGGGTGTCGCTGTTGA-3' |
| **U6** | Forward: 5'-CGCTTCGGCAGCACATATAC-3' |
|  | Reverse: 5'-AAATATGGAACGCTTCACGA-3' |
