# Supplementary material for: Long non-coding RNAs PGM5-AS1 upregulates Decorin (DCN) to inhibit cervical cancer progression by sponging miR-4284
Source: Bioengineered. 2022 Apr 14;13(4):9872–84. doi: 10.1080/21655979.2022.2062088 (PMC9161867; doi:10.1080/21655979.2022.2062088)
Supplement: Supplemental Material [file KBIE_A_2062088_SM1689.zip › supplementary/Supplementary Table 2.docx]

Supplementary Table 2 The miRNAs binding to PGM5-AS1 were predicted by miRDB.

| miRNA Name | Target Score |
| --- | --- |
| hsa-miR-4709-3p | 87 |
| hsa-miR-3065-5p | 84 |
| hsa-miR-452-3p | 79 |
| hsa-miR-4766-3p | 78 |
| hsa-miR-5701 | 76 |
| hsa-miR-3686 | 74 |
| hsa-miR-450b-5p | 71 |
| hsa-miR-587 | 70 |
| hsa-miR-216b-3p | 70 |
| hsa-miR-4695-3p | 63 |
| hsa-miR-4284 | 60 |
| hsa-miR-6743-5p | 58 |
| hsa-miR-4688 | 58 |
| hsa-miR-4471 | 54 |
| hsa-miR-8059 | 52 |
| hsa-miR-627-3p | 52 |
