# Supplementary material for: Long non-coding RNAs PGM5-AS1 upregulates Decorin (DCN) to inhibit cervical cancer progression by sponging miR-4284
Source: Bioengineered. 2022 Apr 14;13(4):9872–84. doi: 10.1080/21655979.2022.2062088 (PMC9161867; doi:10.1080/21655979.2022.2062088)
Supplement: Supplemental Material [file KBIE_A_2062088_SM1689.zip › supplementary/Supplementary Table 3.docx]

Supplementary Table 2 The miRNAs binding to DCN were predicted by miRWalk.

| miRDN | position |
| --- | --- |
| hsa-let-7a-2-3p | 3UTR |
| hsa-let-7b-5p | 3UTR |
| hsa-let-7c-5p | 3UTR |
| hsa-let-7d-5p | 3UTR |
| hsa-let-7e-3p | 3UTR |
| hsa-miR-15a-3p | 3UTR |
| hsa-miR-19b-1-5p | 3UTR |
| hsa-miR-19b-2-5p | 3UTR |
| hsa-miR-21-3p | 3UTR |
| hsa-miR-24-3p | 3UTR |
| hsa-miR-25-5p | 3UTR |
| hsa-miR-25-3p | 3UTR |
| hsa-miR-27a-5p | 3UTR |
| hsa-miR-28-3p | 3UTR |
| hsa-miR-30a-3p | 3UTR |
| hsa-miR-31-5p | 3UTR |
| hsa-miR-92a-2-5p | 3UTR |
| hsa-miR-93-5p | 3UTR |
| hsa-miR-29b-1-5p | 3UTR |
| hsa-miR-29b-3p | 3UTR |
| hsa-miR-103a-2-5p | 3UTR |
| hsa-miR-103a-3p | 3UTR |
| hsa-miR-103a-1-5p | 3UTR |
| hsa-miR-107 | 3UTR |
| hsa-miR-192-3p | 3UTR |
| hsa-miR-197-3p | 3UTR |
| hsa-miR-199a-5p | 3UTR |
| hsa-miR-30c-2-3p | 3UTR |
| hsa-miR-30d-5p | 3UTR |
| hsa-miR-30d-3p | 3UTR |
| hsa-miR-139-3p | 3UTR |
| hsa-miR-147a | 3UTR |
| hsa-miR-7-1-3p | 3UTR |
| hsa-miR-182-5p | 3UTR |
| hsa-miR-182-3p | 3UTR |
| hsa-miR-183-5p | 3UTR |
| hsa-miR-187-5p | 3UTR |
| hsa-miR-187-3p | 3UTR |
| hsa-miR-196a-3p | 3UTR |
| hsa-miR-199b-5p | 3UTR |
| hsa-miR-203a-5p | 3UTR |
| hsa-miR-204-5p | 3UTR |
| hsa-miR-204-3p | 3UTR |
| hsa-miR-205-5p | 3UTR |
| hsa-miR-210-3p | 3UTR |
| hsa-miR-211-5p | 3UTR |
| hsa-miR-212-5p | 3UTR |
| hsa-miR-214-5p | 3UTR |
| hsa-miR-214-3p | 3UTR |
| hsa-miR-218-5p | 3UTR |
| hsa-miR-219a-1-3p | 3UTR |
| hsa-miR-221-5p | 3UTR |
| hsa-miR-221-3p | 3UTR |
| hsa-miR-222-5p | 3UTR |
| hsa-miR-222-3p | 3UTR |
| hsa-miR-200b-5p | 3UTR |
| hsa-let-7i-5p | 3UTR |
| hsa-let-7i-3p | 3UTR |
| hsa-miR-15b-5p | 3UTR |
| hsa-miR-23b-5p | 3UTR |
| hsa-miR-30b-3p | 3UTR |
| hsa-miR-124-5p | 3UTR |
| hsa-miR-124-3p | 3UTR |
| hsa-miR-125b-1-3p | 3UTR |
| hsa-miR-130a-5p | 3UTR |
| hsa-miR-135a-2-3p | 3UTR |
| hsa-miR-137-5p | 3UTR |
| hsa-miR-138-5p | 3UTR |
| hsa-miR-140-5p | 3UTR |
| hsa-miR-142-3p | 3UTR |
| hsa-miR-145-5p | 3UTR |
| hsa-miR-145-3p | 3UTR |
| hsa-miR-191-3p | 3UTR |
| hsa-miR-125b-2-3p | 3UTR |
| hsa-miR-127-5p | 3UTR |
| hsa-miR-127-3p | 3UTR |
| hsa-miR-134-5p | 3UTR |
| hsa-miR-134-3p | 3UTR |
| hsa-miR-150-5p | 3UTR |
| hsa-miR-150-3p | 3UTR |
| hsa-miR-185-3p | 3UTR |
| hsa-miR-188-3p | 3UTR |
| hsa-miR-193a-5p | 3UTR |
| hsa-miR-194-5p | 3UTR |
| hsa-miR-206 | 3UTR |
| hsa-miR-320a-5p | 3UTR |
| hsa-miR-320a-3p | 3UTR |
| hsa-miR-200c-5p | 3UTR |
| hsa-miR-200c-3p | 3UTR |
| hsa-miR-155-5p | 3UTR |
| hsa-miR-128-2-5p | 3UTR |
| hsa-miR-106b-5p | 3UTR |
| hsa-miR-106b-3p | 3UTR |
| hsa-miR-29c-5p | 3UTR |
| hsa-miR-302a-3p | 3UTR |
| hsa-miR-34b-5p | 3UTR |
| hsa-miR-99b-3p | 3UTR |
| hsa-miR-296-5p | 3UTR |
| hsa-miR-130b-3p | 3UTR |
| hsa-miR-362-3p | 3UTR |
| hsa-miR-363-5p | 3UTR |
| hsa-miR-365b-5p | 3UTR |
| hsa-miR-302b-3p | 3UTR |
| hsa-miR-302c-5p | 3UTR |
| hsa-miR-302c-3p | 3UTR |
| hsa-miR-302d-5p | 3UTR |
| hsa-miR-370-3p | 3UTR |
| hsa-miR-371a-3p | 3UTR |
| hsa-miR-372-5p | 3UTR |
| hsa-miR-373-3p | 3UTR |
| hsa-miR-378a-5p | 3UTR |
| hsa-miR-381-5p | 3UTR |
| hsa-miR-381-3p | 3UTR |
| hsa-miR-383-5p | 3UTR |
| hsa-miR-330-3p | 3UTR |
| hsa-miR-337-3p | 3UTR |
| hsa-miR-323a-5p | 3UTR |
| hsa-miR-326 | 3UTR |
| hsa-miR-151a-3p | 3UTR |
| hsa-miR-135b-5p | 3UTR |
| hsa-miR-135b-3p | 3UTR |
| hsa-miR-324-3p | 3UTR |
| hsa-miR-339-3p | 3UTR |
| hsa-miR-335-5p | 3UTR |
| hsa-miR-325 | 3UTR |
| hsa-miR-345-3p | 3UTR |
| hsa-miR-346 | 3UTR |
| hsa-miR-424-5p | 3UTR |
| hsa-miR-424-3p | 3UTR |
| hsa-miR-20b-3p | 3UTR |
| hsa-miR-431-5p | 3UTR |
| hsa-miR-431-3p | 3UTR |
| hsa-miR-433-5p | 3UTR |
| hsa-miR-329-5p | 3UTR |
| hsa-miR-409-3p | 3UTR |
| hsa-miR-412-5p | 3UTR |
| hsa-miR-483-5p | 3UTR |
| hsa-miR-484 | 3UTR |
| hsa-miR-485-5p | 3UTR |
| hsa-miR-485-3p | 3UTR |
| hsa-miR-487a-3p | 3UTR |
| hsa-miR-490-5p | 3UTR |
| hsa-miR-490-3p | 3UTR |
| hsa-miR-146b-5p | 3UTR |
| hsa-miR-146b-3p | 3UTR |
| hsa-miR-492 | 3UTR |
| hsa-miR-493-3p | 3UTR |
| hsa-miR-432-5p | 3UTR |
| hsa-miR-494-5p | 3UTR |
| hsa-miR-495-3p | 3UTR |
| hsa-miR-496 | 3UTR |
| hsa-miR-193b-5p | 3UTR |
| hsa-miR-497-5p | 3UTR |
| hsa-miR-181d-3p | 3UTR |
| hsa-miR-520e-5p | 3UTR |
| hsa-miR-520e-3p | 3UTR |
| hsa-miR-515-5p | 3UTR |
| hsa-miR-515-3p | 3UTR |
| hsa-miR-519e-5p | 3UTR |
| hsa-miR-519e-3p | 3UTR |
| hsa-miR-520f-5p | 3UTR |
| hsa-miR-520f-3p | 3UTR |
| hsa-miR-520a-5p | 3UTR |
| hsa-miR-520a-3p | 3UTR |
| hsa-miR-526b-3p | 3UTR |
| hsa-miR-519b-3p | 3UTR |
| hsa-miR-525-5p | 3UTR |
| hsa-miR-525-3p | 3UTR |
| hsa-miR-520b-3p | 3UTR |
| hsa-miR-518b | 3UTR |
| hsa-miR-526a-3p | 3UTR |
| hsa-miR-520c-3p | 3UTR |
| hsa-miR-518c-3p | 3UTR |
| hsa-miR-524-5p | 3UTR |
| hsa-miR-524-3p | 3UTR |
| hsa-miR-519d-5p | 3UTR |
| hsa-miR-519d-3p | 3UTR |
| hsa-miR-520g-5p | 3UTR |
| hsa-miR-520g-3p | 3UTR |
| hsa-miR-516b-3p | 3UTR |
| hsa-miR-518e-3p | 3UTR |
| hsa-miR-518a-3p | 3UTR |
| hsa-miR-520h | 3UTR |
| hsa-miR-522-3p | 3UTR |
| hsa-miR-516a-3p | 3UTR |
| hsa-miR-500a-5p | 3UTR |
| hsa-miR-501-3p | 3UTR |
| hsa-miR-503-5p | 3UTR |
| hsa-miR-503-3p | 3UTR |
| hsa-miR-504-3p | 3UTR |
| hsa-miR-513a-5p | 3UTR |
| hsa-miR-508-5p | 3UTR |
| hsa-miR-508-3p | 3UTR |
| hsa-miR-509-3p | 3UTR |
| hsa-miR-532-5p | 3UTR |
| hsa-miR-532-3p | 3UTR |
| hsa-miR-455-5p | 3UTR |
| hsa-miR-455-3p | 3UTR |
| hsa-miR-539-5p | 3UTR |
| hsa-miR-487b-3p | 3UTR |
| hsa-miR-554 | 3UTR |
| hsa-miR-92b-5p | 3UTR |
| hsa-miR-563 | 3UTR |
| hsa-miR-564 | 3UTR |
| hsa-miR-551b-5p | 3UTR |
| hsa-miR-570-5p | 3UTR |
| hsa-miR-574-5p | 3UTR |
| hsa-miR-575 | 3UTR |
| hsa-miR-576-3p | 3UTR |
| hsa-miR-581 | 3UTR |
| hsa-miR-583 | 3UTR |
| hsa-miR-584-3p | 3UTR |
| hsa-miR-585-3p | 3UTR |
| hsa-miR-586 | 3UTR |
| hsa-miR-587 | 3UTR |
| hsa-miR-548b-3p | 3UTR |
| hsa-miR-593-3p | 3UTR |
| hsa-miR-595 | 3UTR |
| hsa-miR-596 | 3UTR |
| hsa-miR-597-5p | 3UTR |
| hsa-miR-597-3p | 3UTR |
| hsa-miR-598-5p | 3UTR |
| hsa-miR-602 | 3UTR |
| hsa-miR-603 | 3UTR |
| hsa-miR-604 | 3UTR |
| hsa-miR-605-5p | 3UTR |
| hsa-miR-609 | 3UTR |
| hsa-miR-610 | 3UTR |
| hsa-miR-615-3p | 3UTR |
| hsa-miR-619-5p | 3UTR |
| hsa-miR-623 | 3UTR |
| hsa-miR-627-5p | 3UTR |
| hsa-miR-628-3p | 3UTR |
| hsa-miR-629-5p | 3UTR |
| hsa-miR-631 | 3UTR |
| hsa-miR-33b-3p | 3UTR |
| hsa-miR-632 | 3UTR |
| hsa-miR-637 | 3UTR |
| hsa-miR-639 | 3UTR |
| hsa-miR-640 | 3UTR |
| hsa-miR-641 | 3UTR |
| hsa-miR-642a-5p | 3UTR |
| hsa-miR-646 | 3UTR |
| hsa-miR-649 | 3UTR |
| hsa-miR-650 | 3UTR |
| hsa-miR-651-5p | 3UTR |
| hsa-miR-652-5p | 3UTR |
| hsa-miR-653-3p | 3UTR |
| hsa-miR-654-5p | 3UTR |
| hsa-miR-656-5p | 3UTR |
| hsa-miR-549a-5p | 3UTR |
| hsa-miR-657 | 3UTR |
| hsa-miR-659-5p | 3UTR |
| hsa-miR-660-3p | 3UTR |
| hsa-miR-758-3p | 3UTR |
| hsa-miR-1264 | 3UTR |
| hsa-miR-671-5p | 3UTR |
| hsa-miR-671-3p | 3UTR |
| hsa-miR-668-5p | 3UTR |
| hsa-miR-668-3p | 3UTR |
| hsa-miR-767-5p | 3UTR |
| hsa-miR-1224-5p | 3UTR |
| hsa-miR-1224-3p | 3UTR |
| hsa-miR-320b | 3UTR |
| hsa-miR-320c | 3UTR |
| hsa-miR-1296-5p | 3UTR |
| hsa-miR-1296-3p | 3UTR |
| hsa-miR-1468-5p | 3UTR |
| hsa-miR-1271-3p | 3UTR |
| hsa-miR-1301-3p | 3UTR |
| hsa-miR-454-5p | 3UTR |
| hsa-miR-1185-2-3p | 3UTR |
| hsa-miR-449c-5p | 3UTR |
| hsa-miR-769-5p | 3UTR |
| hsa-miR-1185-1-3p | 3UTR |
| hsa-miR-670-5p | 3UTR |
| hsa-miR-1298-5p | 3UTR |
| hsa-miR-2113 | 3UTR |
| hsa-miR-761 | 3UTR |
| hsa-miR-765 | 3UTR |
| hsa-miR-770-5p | 3UTR |
| hsa-miR-675-5p | 3UTR |
| hsa-miR-298 | 3UTR |
| hsa-miR-891a-5p | 3UTR |
| hsa-miR-891a-3p | 3UTR |
| hsa-miR-300 | 3UTR |
| hsa-miR-892a | 3UTR |
| hsa-miR-450b-5p | 3UTR |
| hsa-miR-888-3p | 3UTR |
| hsa-miR-541-5p | 3UTR |
| hsa-miR-889-5p | 3UTR |
| hsa-miR-876-5p | 3UTR |
| hsa-miR-744-5p | 3UTR |
| hsa-miR-885-3p | 3UTR |
| hsa-miR-877-5p | 3UTR |
| hsa-miR-877-3p | 3UTR |
| hsa-miR-887-3p | 3UTR |
| hsa-miR-665 | 3UTR |
| hsa-miR-873-5p | 3UTR |
| hsa-miR-873-3p | 3UTR |
| hsa-miR-301b-5p | 3UTR |
| hsa-miR-216b-5p | 3UTR |
| hsa-miR-922 | 3UTR |
| hsa-miR-509-3-5p | 3UTR |
| hsa-miR-936 | 3UTR |
| hsa-miR-937-5p | 3UTR |
| hsa-miR-937-3p | 3UTR |
| hsa-miR-941 | 3UTR |
| hsa-miR-942-3p | 3UTR |
| hsa-miR-297 | 3UTR |
| hsa-miR-1178-5p | 3UTR |
| hsa-miR-1180-3p | 3UTR |
| hsa-miR-1181 | 3UTR |
| hsa-miR-1183 | 3UTR |
| hsa-miR-1227-5p | 3UTR |
| hsa-miR-1227-3p | 3UTR |
| hsa-miR-1228-5p | 3UTR |
| hsa-miR-1229-3p | 3UTR |
| hsa-miR-1233-5p | 3UTR |
| hsa-miR-1233-3p | 3UTR |
| hsa-miR-1236-5p | 3UTR |
| hsa-miR-1236-3p | 3UTR |
| hsa-miR-1238-5p | 3UTR |
| hsa-miR-1238-3p | 3UTR |
| hsa-miR-1202 | 3UTR |
| hsa-miR-1203 | 3UTR |
| hsa-miR-663b | 3UTR |
| hsa-miR-1204 | 3UTR |
| hsa-miR-1205 | 3UTR |
| hsa-miR-1285-3p | 3UTR |
| hsa-miR-1286 | 3UTR |
| hsa-miR-1287-5p | 3UTR |
| hsa-miR-1287-3p | 3UTR |
| hsa-miR-1289 | 3UTR |
| hsa-miR-1290 | 3UTR |
| hsa-miR-1293 | 3UTR |
| hsa-miR-1295a | 3UTR |
| hsa-miR-1299 | 3UTR |
| hsa-miR-548l | 3UTR |
| hsa-miR-1303 | 3UTR |
| hsa-miR-1304-5p | 3UTR |
| hsa-miR-1304-3p | 3UTR |
| hsa-miR-1244 | 3UTR |
| hsa-miR-1246 | 3UTR |
| hsa-miR-1249-3p | 3UTR |
| hsa-miR-1250-5p | 3UTR |
| hsa-miR-1256 | 3UTR |
| hsa-miR-1257 | 3UTR |
| hsa-miR-1260a | 3UTR |
| hsa-miR-1262 | 3UTR |
| hsa-miR-1263 | 3UTR |
| hsa-miR-1265 | 3UTR |
| hsa-miR-1266-5p | 3UTR |
| hsa-miR-1267 | 3UTR |
| hsa-miR-1275 | 3UTR |
| hsa-miR-302e | 3UTR |
| hsa-miR-1281 | 3UTR |
| hsa-miR-1288-5p | 3UTR |
| hsa-miR-1288-3p | 3UTR |
| hsa-miR-1292-5p | 3UTR |
| hsa-miR-1255b-5p | 3UTR |
| hsa-miR-1255b-2-3p | 3UTR |
| hsa-miR-1306-5p | 3UTR |
| hsa-miR-1307-5p | 3UTR |
| hsa-miR-513b-5p | 3UTR |
| hsa-miR-513b-3p | 3UTR |
| hsa-miR-1321 | 3UTR |
| hsa-miR-1470 | 3UTR |
| hsa-miR-320d | 3UTR |
| hsa-miR-1825 | 3UTR |
| hsa-miR-1827 | 3UTR |
| hsa-miR-1909-5p | 3UTR |
| hsa-miR-1910-5p | 3UTR |
| hsa-miR-1910-3p | 3UTR |
| hsa-miR-1912-5p | 3UTR |
| hsa-miR-1912-3p | 3UTR |
| hsa-miR-1915-5p | 3UTR |
| hsa-miR-1915-3p | 3UTR |
| hsa-miR-1976 | 3UTR |
| hsa-miR-2114-5p | 3UTR |
| hsa-miR-2114-3p | 3UTR |
| hsa-miR-2116-5p | 3UTR |
| hsa-miR-2117 | 3UTR |
| hsa-miR-548q | 3UTR |
| hsa-miR-2276-5p | 3UTR |
| hsa-miR-2276-3p | 3UTR |
| hsa-miR-2277-5p | 3UTR |
| hsa-miR-2682-3p | 3UTR |
| hsa-miR-718 | 3UTR |
| hsa-miR-3120-5p | 3UTR |
| hsa-miR-3120-3p | 3UTR |
| hsa-miR-3121-3p | 3UTR |
| hsa-miR-3122 | 3UTR |
| hsa-miR-3130-5p | 3UTR |
| hsa-miR-3130-3p | 3UTR |
| hsa-miR-3131 | 3UTR |
| hsa-miR-3132 | 3UTR |
| hsa-miR-378b | 3UTR |
| hsa-miR-3134 | 3UTR |
| hsa-miR-3135a | 3UTR |
| hsa-miR-544b | 3UTR |
| hsa-miR-3137 | 3UTR |
| hsa-miR-3139 | 3UTR |
| hsa-miR-3142 | 3UTR |
| hsa-miR-3144-5p | 3UTR |
| hsa-miR-3147 | 3UTR |
| hsa-miR-548v | 3UTR |
| hsa-miR-3149 | 3UTR |
| hsa-miR-3150a-5p | 3UTR |
| hsa-miR-3152-3p | 3UTR |
| hsa-miR-3153 | 3UTR |
| hsa-miR-3074-5p | 3UTR |
| hsa-miR-3154 | 3UTR |
| hsa-miR-3156-5p | 3UTR |
| hsa-miR-3156-3p | 3UTR |
| hsa-miR-3157-3p | 3UTR |
| hsa-miR-3158-3p | 3UTR |
| hsa-miR-3160-3p | 3UTR |
| hsa-miR-3161 | 3UTR |
| hsa-miR-3162-5p | 3UTR |
| hsa-miR-3162-3p | 3UTR |
| hsa-miR-3164 | 3UTR |
| hsa-miR-3170 | 3UTR |
| hsa-miR-3173-5p | 3UTR |
| hsa-miR-3173-3p | 3UTR |
| hsa-miR-3174 | 3UTR |
| hsa-miR-3181 | 3UTR |
| hsa-miR-3183 | 3UTR |
| hsa-miR-3185 | 3UTR |
| hsa-miR-3065-3p | 3UTR |
| hsa-miR-3186-3p | 3UTR |
| hsa-miR-3188 | 3UTR |
| hsa-miR-3190-5p | 3UTR |
| hsa-miR-3192-5p | 3UTR |
| hsa-miR-3193 | 3UTR |
| hsa-miR-3197 | 3UTR |
| hsa-miR-3199 | 3UTR |
| hsa-miR-3200-5p | 3UTR |
| hsa-miR-514b-5p | 3UTR |
| hsa-miR-514b-3p | 3UTR |
| hsa-miR-3202 | 3UTR |
| hsa-miR-4297 | 3UTR |
| hsa-miR-4301 | 3UTR |
| hsa-miR-4299 | 3UTR |
| hsa-miR-4298 | 3UTR |
| hsa-miR-4306 | 3UTR |
| hsa-miR-4310 | 3UTR |
| hsa-miR-4322 | 3UTR |
| hsa-miR-4324 | 3UTR |
| hsa-miR-4257 | 3UTR |
| hsa-miR-4258 | 3UTR |
| hsa-miR-4254 | 3UTR |
| hsa-miR-4255 | 3UTR |
| hsa-miR-4327 | 3UTR |
| hsa-miR-4270 | 3UTR |
| hsa-miR-4271 | 3UTR |
| hsa-miR-4277 | 3UTR |
| hsa-miR-4279 | 3UTR |
| hsa-miR-4280 | 3UTR |
| hsa-miR-4289 | 3UTR |
| hsa-miR-4290 | 3UTR |
| hsa-miR-4291 | 3UTR |
| hsa-miR-3605-3p | 3UTR |
| hsa-miR-3606-5p | 3UTR |
| hsa-miR-3612 | 3UTR |
| hsa-miR-3614-5p | 3UTR |
| hsa-miR-3614-3p | 3UTR |
| hsa-miR-3616-5p | 3UTR |
| hsa-miR-3616-3p | 3UTR |
| hsa-miR-3617-5p | 3UTR |
| hsa-miR-3617-3p | 3UTR |
| hsa-miR-3619-5p | 3UTR |
| hsa-miR-3619-3p | 3UTR |
| hsa-miR-3621 | 3UTR |
| hsa-miR-3650 | 3UTR |
| hsa-miR-3651 | 3UTR |
| hsa-miR-3652 | 3UTR |
| hsa-miR-3655 | 3UTR |
| hsa-miR-3657 | 3UTR |
| hsa-miR-3658 | 3UTR |
| hsa-miR-3659 | 3UTR |
| hsa-miR-3660 | 3UTR |
| hsa-miR-3663-3p | 3UTR |
| hsa-miR-3664-3p | 3UTR |
| hsa-miR-3667-5p | 3UTR |
| hsa-miR-3667-3p | 3UTR |
| hsa-miR-3670 | 3UTR |
| hsa-miR-3678-3p | 3UTR |
| hsa-miR-3679-3p | 3UTR |
| hsa-miR-3680-5p | 3UTR |
| hsa-miR-3680-3p | 3UTR |
| hsa-miR-3681-5p | 3UTR |
| hsa-miR-3681-3p | 3UTR |
| hsa-miR-3683 | 3UTR |
| hsa-miR-3684 | 3UTR |
| hsa-miR-3685 | 3UTR |
| hsa-miR-3686 | 3UTR |
| hsa-miR-3688-5p | 3UTR |
| hsa-miR-3689a-5p | 3UTR |
| hsa-miR-3689a-3p | 3UTR |
| hsa-miR-3690 | 3UTR |
| hsa-miR-3691-5p | 3UTR |
| hsa-miR-3691-3p | 3UTR |
| hsa-miR-3692-5p | 3UTR |
| hsa-miR-3692-3p | 3UTR |
| hsa-miR-3714 | 3UTR |
| hsa-miR-3180 | 3UTR |
| hsa-miR-3689b-5p | 3UTR |
| hsa-miR-3689b-3p | 3UTR |
| hsa-miR-3911 | 3UTR |
| hsa-miR-3913-5p | 3UTR |
| hsa-miR-3913-3p | 3UTR |
| hsa-miR-3917 | 3UTR |
| hsa-miR-3918 | 3UTR |
| hsa-miR-3919 | 3UTR |
| hsa-miR-3150b-5p | 3UTR |
| hsa-miR-3150b-3p | 3UTR |
| hsa-miR-3926 | 3UTR |
| hsa-miR-3927-3p | 3UTR |
| hsa-miR-676-3p | 3UTR |
| hsa-miR-3928-5p | 3UTR |
| hsa-miR-3934-5p | 3UTR |
| hsa-miR-3934-3p | 3UTR |
| hsa-miR-3937 | 3UTR |
| hsa-miR-3938 | 3UTR |
| hsa-miR-3939 | 3UTR |
| hsa-miR-3943 | 3UTR |
| hsa-miR-642b-5p | 3UTR |
| hsa-miR-550b-2-5p | 3UTR |
| hsa-miR-1268b | 3UTR |
| hsa-miR-378e | 3UTR |
| hsa-miR-4421 | 3UTR |
| hsa-miR-4423-5p | 3UTR |
| hsa-miR-4423-3p | 3UTR |
| hsa-miR-4425 | 3UTR |
| hsa-miR-4427 | 3UTR |
| hsa-miR-4428 | 3UTR |
| hsa-miR-4429 | 3UTR |
| hsa-miR-4430 | 3UTR |
| hsa-miR-4433a-5p | 3UTR |
| hsa-miR-4435 | 3UTR |
| hsa-miR-4436a | 3UTR |
| hsa-miR-4438 | 3UTR |
| hsa-miR-4439 | 3UTR |
| hsa-miR-4441 | 3UTR |
| hsa-miR-4446-5p | 3UTR |
| hsa-miR-4446-3p | 3UTR |
| hsa-miR-4449 | 3UTR |
| hsa-miR-4450 | 3UTR |
| hsa-miR-548ah-3p | 3UTR |
| hsa-miR-4451 | 3UTR |
| hsa-miR-4453 | 3UTR |
| hsa-miR-4454 | 3UTR |
| hsa-miR-4455 | 3UTR |
| hsa-miR-4458 | 3UTR |
| hsa-miR-3135b | 3UTR |
| hsa-miR-4462 | 3UTR |
| hsa-miR-4463 | 3UTR |
| hsa-miR-548ai | 3UTR |
| hsa-miR-4467 | 3UTR |
| hsa-miR-4468 | 3UTR |
| hsa-miR-4472 | 3UTR |
| hsa-miR-4473 | 3UTR |
| hsa-miR-4475 | 3UTR |
| hsa-miR-4476 | 3UTR |
| hsa-miR-4478 | 3UTR |
| hsa-miR-3689c | 3UTR |
| hsa-miR-3689d | 3UTR |
| hsa-miR-3689e | 3UTR |
| hsa-miR-3155b | 3UTR |
| hsa-miR-4482-5p | 3UTR |
| hsa-miR-4482-3p | 3UTR |
| hsa-miR-4483 | 3UTR |
| hsa-miR-4484 | 3UTR |
| hsa-miR-4487 | 3UTR |
| hsa-miR-4489 | 3UTR |
| hsa-miR-4490 | 3UTR |
| hsa-miR-4491 | 3UTR |
| hsa-miR-4493 | 3UTR |
| hsa-miR-4496 | 3UTR |
| hsa-miR-4499 | 3UTR |
| hsa-miR-4503 | 3UTR |
| hsa-miR-4504 | 3UTR |
| hsa-miR-4506 | 3UTR |
| hsa-miR-2392 | 3UTR |
| hsa-miR-4510 | 3UTR |
| hsa-miR-4511 | 3UTR |
| hsa-miR-4513 | 3UTR |
| hsa-miR-4516 | 3UTR |
| hsa-miR-4518 | 3UTR |
| hsa-miR-4520-5p | 3UTR |
| hsa-miR-4520-3p | 3UTR |
| hsa-miR-1269b | 3UTR |
| hsa-miR-4523 | 3UTR |
| hsa-miR-4524a-5p | 3UTR |
| hsa-miR-4525 | 3UTR |
| hsa-miR-4534 | 3UTR |
| hsa-miR-4537 | 3UTR |
| hsa-miR-4538 | 3UTR |
| hsa-miR-3974 | 3UTR |
| hsa-miR-3975 | 3UTR |
| hsa-miR-3976 | 3UTR |
| hsa-miR-4632-5p | 3UTR |
| hsa-miR-4632-3p | 3UTR |
| hsa-miR-4633-3p | 3UTR |
| hsa-miR-4638-5p | 3UTR |
| hsa-miR-4640-5p | 3UTR |
| hsa-miR-4640-3p | 3UTR |
| hsa-miR-4644 | 3UTR |
| hsa-miR-4645-3p | 3UTR |
| hsa-miR-4646-5p | 3UTR |
| hsa-miR-4646-3p | 3UTR |
| hsa-miR-4649-3p | 3UTR |
| hsa-miR-4650-5p | 3UTR |
| hsa-miR-4650-3p | 3UTR |
| hsa-miR-4653-5p | 3UTR |
| hsa-miR-4654 | 3UTR |
| hsa-miR-4656 | 3UTR |
| hsa-miR-4657 | 3UTR |
| hsa-miR-4658 | 3UTR |
| hsa-miR-4660 | 3UTR |
| hsa-miR-4661-5p | 3UTR |
| hsa-miR-4661-3p | 3UTR |
| hsa-miR-4663 | 3UTR |
| hsa-miR-4664-5p | 3UTR |
| hsa-miR-4666a-3p | 3UTR |
| hsa-miR-4670-3p | 3UTR |
| hsa-miR-4671-5p | 3UTR |
| hsa-miR-4672 | 3UTR |
| hsa-miR-4673 | 3UTR |
| hsa-miR-4674 | 3UTR |
| hsa-miR-4675 | 3UTR |
| hsa-miR-4677-3p | 3UTR |
| hsa-miR-4679 | 3UTR |
| hsa-miR-4681 | 3UTR |
| hsa-miR-4684-5p | 3UTR |
| hsa-miR-4685-5p | 3UTR |
| hsa-miR-4685-3p | 3UTR |
| hsa-miR-4686 | 3UTR |
| hsa-miR-4687-5p | 3UTR |
| hsa-miR-4688 | 3UTR |
| hsa-miR-4689 | 3UTR |
| hsa-miR-4690-5p | 3UTR |
| hsa-miR-4690-3p | 3UTR |
| hsa-miR-4691-3p | 3UTR |
| hsa-miR-4692 | 3UTR |
| hsa-miR-4694-5p | 3UTR |
| hsa-miR-4694-3p | 3UTR |
| hsa-miR-4695-5p | 3UTR |
| hsa-miR-4695-3p | 3UTR |
| hsa-miR-4697-5p | 3UTR |
| hsa-miR-4698 | 3UTR |
| hsa-miR-4699-5p | 3UTR |
| hsa-miR-4700-5p | 3UTR |
| hsa-miR-4701-3p | 3UTR |
| hsa-miR-4704-5p | 3UTR |
| hsa-miR-4704-3p | 3UTR |
| hsa-miR-4706 | 3UTR |
| hsa-miR-4708-5p | 3UTR |
| hsa-miR-4708-3p | 3UTR |
| hsa-miR-4709-3p | 3UTR |
| hsa-miR-203b-3p | 3UTR |
| hsa-miR-4711-5p | 3UTR |
| hsa-miR-4711-3p | 3UTR |
| hsa-miR-4714-3p | 3UTR |
| hsa-miR-4715-5p | 3UTR |
| hsa-miR-4715-3p | 3UTR |
| hsa-miR-4716-5p | 3UTR |
| hsa-miR-4716-3p | 3UTR |
| hsa-miR-4717-5p | 3UTR |
| hsa-miR-4721 | 3UTR |
| hsa-miR-4722-5p | 3UTR |
| hsa-miR-4723-5p | 3UTR |
| hsa-miR-4724-3p | 3UTR |
| hsa-miR-4725-5p | 3UTR |
| hsa-miR-4725-3p | 3UTR |
| hsa-miR-4727-3p | 3UTR |
| hsa-miR-4728-5p | 3UTR |
| hsa-miR-4732-5p | 3UTR |
| hsa-miR-4732-3p | 3UTR |
| hsa-miR-4733-3p | 3UTR |
| hsa-miR-4734 | 3UTR |
| hsa-miR-4736 | 3UTR |
| hsa-miR-4737 | 3UTR |
| hsa-miR-3064-5p | 3UTR |
| hsa-miR-4740-3p | 3UTR |
| hsa-miR-4743-5p | 3UTR |
| hsa-miR-4744 | 3UTR |
| hsa-miR-122b-5p | 3UTR |
| hsa-miR-4745-3p | 3UTR |
| hsa-miR-4747-3p | 3UTR |
| hsa-miR-4748 | 3UTR |
| hsa-miR-4750-3p | 3UTR |
| hsa-miR-4753-5p | 3UTR |
| hsa-miR-371b-3p | 3UTR |
| hsa-miR-4755-3p | 3UTR |
| hsa-miR-499b-5p | 3UTR |
| hsa-miR-4756-5p | 3UTR |
| hsa-miR-4756-3p | 3UTR |
| hsa-miR-4757-5p | 3UTR |
| hsa-miR-4757-3p | 3UTR |
| hsa-miR-4760-5p | 3UTR |
| hsa-miR-4761-3p | 3UTR |
| hsa-miR-4764-5p | 3UTR |
| hsa-miR-4767 | 3UTR |
| hsa-miR-4768-3p | 3UTR |
| hsa-miR-4769-5p | 3UTR |
| hsa-miR-4773 | 3UTR |
| hsa-miR-4774-3p | 3UTR |
| hsa-miR-4776-5p | 3UTR |
| hsa-miR-4776-3p | 3UTR |
| hsa-miR-4777-3p | 3UTR |
| hsa-miR-4778-5p | 3UTR |
| hsa-miR-4778-3p | 3UTR |
| hsa-miR-4436b-5p | 3UTR |
| hsa-miR-4436b-3p | 3UTR |
| hsa-miR-4784 | 3UTR |
| hsa-miR-4785 | 3UTR |
| hsa-miR-2467-3p | 3UTR |
| hsa-miR-4786-5p | 3UTR |
| hsa-miR-4786-3p | 3UTR |
| hsa-miR-4787-5p | 3UTR |
| hsa-miR-4787-3p | 3UTR |
| hsa-miR-4793-3p | 3UTR |
| hsa-miR-4799-5p | 3UTR |
| hsa-miR-4800-5p | 3UTR |
| hsa-miR-5001-3p | 3UTR |
| hsa-miR-5002-3p | 3UTR |
| hsa-miR-5004-5p | 3UTR |
| hsa-miR-5006-5p | 3UTR |
| hsa-miR-5009-5p | 3UTR |
| hsa-miR-5010-5p | 3UTR |
| hsa-miR-5010-3p | 3UTR |
| hsa-miR-5047 | 3UTR |
| hsa-miR-5087 | 3UTR |
| hsa-miR-5089-3p | 3UTR |
| hsa-miR-5091 | 3UTR |
| hsa-miR-5093 | 3UTR |
| hsa-miR-5189-3p | 3UTR |
| hsa-miR-5192 | 3UTR |
| hsa-miR-5193 | 3UTR |
| hsa-miR-5196-3p | 3UTR |
| hsa-miR-4524b-5p | 3UTR |
| hsa-miR-5100 | 3UTR |
| hsa-miR-5572 | 3UTR |
| hsa-miR-548ar-3p | 3UTR |
| hsa-miR-664b-3p | 3UTR |
| hsa-miR-5580-5p | 3UTR |
| hsa-miR-5584-5p | 3UTR |
| hsa-miR-5584-3p | 3UTR |
| hsa-miR-5586-5p | 3UTR |
| hsa-miR-548au-3p | 3UTR |
| hsa-miR-5589-3p | 3UTR |
| hsa-miR-5680 | 3UTR |
| hsa-miR-5682 | 3UTR |
| hsa-miR-5683 | 3UTR |
| hsa-miR-5684 | 3UTR |
| hsa-miR-5681b | 3UTR |
| hsa-miR-5689 | 3UTR |
| hsa-miR-5691 | 3UTR |
| hsa-miR-5699-3p | 3UTR |
| hsa-miR-5705 | 3UTR |
| hsa-miR-5708 | 3UTR |
| hsa-miR-5739 | 3UTR |
| hsa-miR-1199-3p | 3UTR |
| hsa-miR-6068 | 3UTR |
| hsa-miR-6071 | 3UTR |
| hsa-miR-6072 | 3UTR |
| hsa-miR-6074 | 3UTR |
| hsa-miR-6076 | 3UTR |
| hsa-miR-6078 | 3UTR |
| hsa-miR-6079 | 3UTR |
| hsa-miR-6080 | 3UTR |
| hsa-miR-6083 | 3UTR |
| hsa-miR-6086 | 3UTR |
| hsa-miR-6088 | 3UTR |
| hsa-miR-6090 | 3UTR |
| hsa-miR-6124 | 3UTR |
| hsa-miR-6127 | 3UTR |
| hsa-miR-6129 | 3UTR |
| hsa-miR-6130 | 3UTR |
| hsa-miR-6131 | 3UTR |
| hsa-miR-6133 | 3UTR |
| hsa-miR-6134 | 3UTR |
| hsa-miR-6165 | 3UTR |
| hsa-miR-6499-5p | 3UTR |
| hsa-miR-6499-3p | 3UTR |
| hsa-miR-6500-3p | 3UTR |
| hsa-miR-6501-5p | 3UTR |
| hsa-miR-6502-3p | 3UTR |
| hsa-miR-6503-5p | 3UTR |
| hsa-miR-6503-3p | 3UTR |
| hsa-miR-6505-5p | 3UTR |
| hsa-miR-6508-5p | 3UTR |
| hsa-miR-6508-3p | 3UTR |
| hsa-miR-6509-5p | 3UTR |
| hsa-miR-6509-3p | 3UTR |
| hsa-miR-6510-5p | 3UTR |
| hsa-miR-6510-3p | 3UTR |
| hsa-miR-6511a-5p | 3UTR |
| hsa-miR-6514-5p | 3UTR |
| hsa-miR-6515-5p | 3UTR |
| hsa-miR-6715a-3p | 3UTR |
| hsa-miR-6715b-3p | 3UTR |
| hsa-miR-6717-5p | 3UTR |
| hsa-miR-6511b-5p | 3UTR |
| hsa-miR-6511b-3p | 3UTR |
| hsa-miR-6720-3p | 3UTR |
| hsa-miR-6721-5p | 3UTR |
| hsa-miR-6722-3p | 3UTR |
| hsa-miR-6726-5p | 3UTR |
| hsa-miR-6728-5p | 3UTR |
| hsa-miR-6728-3p | 3UTR |
| hsa-miR-6730-5p | 3UTR |
| hsa-miR-6731-5p | 3UTR |
| hsa-miR-6732-5p | 3UTR |
| hsa-miR-6732-3p | 3UTR |
| hsa-miR-6733-3p | 3UTR |
| hsa-miR-6734-5p | 3UTR |
| hsa-miR-6735-3p | 3UTR |
| hsa-miR-6736-3p | 3UTR |
| hsa-miR-6737-5p | 3UTR |
| hsa-miR-6737-3p | 3UTR |
| hsa-miR-6738-5p | 3UTR |
| hsa-miR-6739-5p | 3UTR |
| hsa-miR-6739-3p | 3UTR |
| hsa-miR-6740-5p | 3UTR |
| hsa-miR-6740-3p | 3UTR |
| hsa-miR-6742-5p | 3UTR |
| hsa-miR-6742-3p | 3UTR |
| hsa-miR-6743-3p | 3UTR |
| hsa-miR-6744-3p | 3UTR |
| hsa-miR-6745 | 3UTR |
| hsa-miR-6746-3p | 3UTR |
| hsa-miR-6747-5p | 3UTR |
| hsa-miR-6747-3p | 3UTR |
| hsa-miR-6748-5p | 3UTR |
| hsa-miR-6748-3p | 3UTR |
| hsa-miR-6750-3p | 3UTR |
| hsa-miR-6751-5p | 3UTR |
| hsa-miR-6751-3p | 3UTR |
| hsa-miR-6752-5p | 3UTR |
| hsa-miR-6754-5p | 3UTR |
| hsa-miR-6754-3p | 3UTR |
| hsa-miR-6755-3p | 3UTR |
| hsa-miR-6756-5p | 3UTR |
| hsa-miR-6756-3p | 3UTR |
| hsa-miR-6758-5p | 3UTR |
| hsa-miR-6759-3p | 3UTR |
| hsa-miR-6760-5p | 3UTR |
| hsa-miR-6761-5p | 3UTR |
| hsa-miR-6762-5p | 3UTR |
| hsa-miR-6762-3p | 3UTR |
| hsa-miR-6763-3p | 3UTR |
| hsa-miR-6764-3p | 3UTR |
| hsa-miR-6766-3p | 3UTR |
| hsa-miR-6767-3p | 3UTR |
| hsa-miR-6769a-3p | 3UTR |
| hsa-miR-6770-5p | 3UTR |
| hsa-miR-6770-3p | 3UTR |
| hsa-miR-6773-5p | 3UTR |
| hsa-miR-6773-3p | 3UTR |
| hsa-miR-6774-5p | 3UTR |
| hsa-miR-6774-3p | 3UTR |
| hsa-miR-6776-5p | 3UTR |
| hsa-miR-6776-3p | 3UTR |
| hsa-miR-6777-5p | 3UTR |
| hsa-miR-6778-5p | 3UTR |
| hsa-miR-6778-3p | 3UTR |
| hsa-miR-6779-5p | 3UTR |
| hsa-miR-6779-3p | 3UTR |
| hsa-miR-6780a-5p | 3UTR |
| hsa-miR-6781-3p | 3UTR |
| hsa-miR-6782-5p | 3UTR |
| hsa-miR-6782-3p | 3UTR |
| hsa-miR-6783-3p | 3UTR |
| hsa-miR-6784-3p | 3UTR |
| hsa-miR-6785-5p | 3UTR |
| hsa-miR-6785-3p | 3UTR |
| hsa-miR-6787-5p | 3UTR |
| hsa-miR-6787-3p | 3UTR |
| hsa-miR-6788-5p | 3UTR |
| hsa-miR-6789-3p | 3UTR |
| hsa-miR-6790-5p | 3UTR |
| hsa-miR-6790-3p | 3UTR |
| hsa-miR-6791-3p | 3UTR |
| hsa-miR-6793-5p | 3UTR |
| hsa-miR-6794-5p | 3UTR |
| hsa-miR-6795-5p | 3UTR |
| hsa-miR-6797-5p | 3UTR |
| hsa-miR-6798-3p | 3UTR |
| hsa-miR-6799-5p | 3UTR |
| hsa-miR-6799-3p | 3UTR |
| hsa-miR-6801-5p | 3UTR |
| hsa-miR-6803-3p | 3UTR |
| hsa-miR-6804-5p | 3UTR |
| hsa-miR-6804-3p | 3UTR |
| hsa-miR-6805-5p | 3UTR |
| hsa-miR-6807-5p | 3UTR |
| hsa-miR-6807-3p | 3UTR |
| hsa-miR-6808-3p | 3UTR |
| hsa-miR-6809-3p | 3UTR |
| hsa-miR-6810-5p | 3UTR |
| hsa-miR-6810-3p | 3UTR |
| hsa-miR-6811-5p | 3UTR |
| hsa-miR-6811-3p | 3UTR |
| hsa-miR-6812-5p | 3UTR |
| hsa-miR-6813-5p | 3UTR |
| hsa-miR-6814-5p | 3UTR |
| hsa-miR-6815-5p | 3UTR |
| hsa-miR-6816-5p | 3UTR |
| hsa-miR-6816-3p | 3UTR |
| hsa-miR-6818-5p | 3UTR |
| hsa-miR-6818-3p | 3UTR |
| hsa-miR-6819-5p | 3UTR |
| hsa-miR-6819-3p | 3UTR |
| hsa-miR-6820-3p | 3UTR |
| hsa-miR-6821-5p | 3UTR |
| hsa-miR-6821-3p | 3UTR |
| hsa-miR-6822-3p | 3UTR |
| hsa-miR-6824-3p | 3UTR |
| hsa-miR-6825-5p | 3UTR |
| hsa-miR-6825-3p | 3UTR |
| hsa-miR-6826-5p | 3UTR |
| hsa-miR-6827-5p | 3UTR |
| hsa-miR-6829-5p | 3UTR |
| hsa-miR-6829-3p | 3UTR |
| hsa-miR-6830-5p | 3UTR |
| hsa-miR-6830-3p | 3UTR |
| hsa-miR-6831-5p | 3UTR |
| hsa-miR-6832-5p | 3UTR |
| hsa-miR-6833-5p | 3UTR |
| hsa-miR-6835-3p | 3UTR |
| hsa-miR-6780b-3p | 3UTR |
| hsa-miR-6836-5p | 3UTR |
| hsa-miR-6836-3p | 3UTR |
| hsa-miR-6837-5p | 3UTR |
| hsa-miR-6837-3p | 3UTR |
| hsa-miR-6838-5p | 3UTR |
| hsa-miR-6838-3p | 3UTR |
| hsa-miR-6839-3p | 3UTR |
| hsa-miR-6840-5p | 3UTR |
| hsa-miR-6841-3p | 3UTR |
| hsa-miR-6842-5p | 3UTR |
| hsa-miR-6843-3p | 3UTR |
| hsa-miR-6845-5p | 3UTR |
| hsa-miR-6846-5p | 3UTR |
| hsa-miR-6846-3p | 3UTR |
| hsa-miR-6847-5p | 3UTR |
| hsa-miR-6849-5p | 3UTR |
| hsa-miR-6850-3p | 3UTR |
| hsa-miR-6853-5p | 3UTR |
| hsa-miR-6854-5p | 3UTR |
| hsa-miR-6855-5p | 3UTR |
| hsa-miR-6856-5p | 3UTR |
| hsa-miR-6857-5p | 3UTR |
| hsa-miR-6857-3p | 3UTR |
| hsa-miR-6858-5p | 3UTR |
| hsa-miR-6769b-5p | 3UTR |
| hsa-miR-6769b-3p | 3UTR |
| hsa-miR-6861-3p | 3UTR |
| hsa-miR-6863 | 3UTR |
| hsa-miR-6864-5p | 3UTR |
| hsa-miR-6864-3p | 3UTR |
| hsa-miR-6865-5p | 3UTR |
| hsa-miR-6865-3p | 3UTR |
| hsa-miR-6869-5p | 3UTR |
| hsa-miR-6869-3p | 3UTR |
| hsa-miR-6870-5p | 3UTR |
| hsa-miR-6870-3p | 3UTR |
| hsa-miR-6872-5p | 3UTR |
| hsa-miR-6872-3p | 3UTR |
| hsa-miR-6873-5p | 3UTR |
| hsa-miR-6873-3p | 3UTR |
| hsa-miR-6874-5p | 3UTR |
| hsa-miR-6874-3p | 3UTR |
| hsa-miR-6875-3p | 3UTR |
| hsa-miR-6876-5p | 3UTR |
| hsa-miR-6876-3p | 3UTR |
| hsa-miR-6878-3p | 3UTR |
| hsa-miR-6879-5p | 3UTR |
| hsa-miR-6880-5p | 3UTR |
| hsa-miR-6880-3p | 3UTR |
| hsa-miR-6881-5p | 3UTR |
| hsa-miR-6882-5p | 3UTR |
| hsa-miR-6882-3p | 3UTR |
| hsa-miR-6883-5p | 3UTR |
| hsa-miR-6884-5p | 3UTR |
| hsa-miR-6886-5p | 3UTR |
| hsa-miR-6887-5p | 3UTR |
| hsa-miR-6888-5p | 3UTR |
| hsa-miR-6889-5p | 3UTR |
| hsa-miR-6890-5p | 3UTR |
| hsa-miR-6891-5p | 3UTR |
| hsa-miR-6891-3p | 3UTR |
| hsa-miR-6892-3p | 3UTR |
| hsa-miR-6893-3p | 3UTR |
| hsa-miR-6894-5p | 3UTR |
| hsa-miR-6895-5p | 3UTR |
| hsa-miR-7106-5p | 3UTR |
| hsa-miR-7106-3p | 3UTR |
| hsa-miR-7108-5p | 3UTR |
| hsa-miR-7108-3p | 3UTR |
| hsa-miR-7109-5p | 3UTR |
| hsa-miR-7110-5p | 3UTR |
| hsa-miR-7110-3p | 3UTR |
| hsa-miR-7111-5p | 3UTR |
| hsa-miR-7112-5p | 3UTR |
| hsa-miR-7113-5p | 3UTR |
| hsa-miR-7113-3p | 3UTR |
| hsa-miR-7114-3p | 3UTR |
| hsa-miR-7150 | 3UTR |
| hsa-miR-7154-3p | 3UTR |
| hsa-miR-7155-5p | 3UTR |
| hsa-miR-7157-5p | 3UTR |
| hsa-miR-7158-3p | 3UTR |
| hsa-miR-7160-5p | 3UTR |
| hsa-miR-7160-3p | 3UTR |
| hsa-miR-7162-5p | 3UTR |
| hsa-miR-7162-3p | 3UTR |
| hsa-miR-7703 | 3UTR |
| hsa-miR-7704 | 3UTR |
| hsa-miR-7843-5p | 3UTR |
| hsa-miR-7843-3p | 3UTR |
| hsa-miR-4433b-3p | 3UTR |
| hsa-miR-1273h-5p | 3UTR |
| hsa-miR-7844-5p | 3UTR |
| hsa-miR-7847-3p | 3UTR |
| hsa-miR-7848-3p | 3UTR |
| hsa-miR-7849-3p | 3UTR |
| hsa-miR-7850-5p | 3UTR |
| hsa-miR-7852-3p | 3UTR |
| hsa-miR-7853-5p | 3UTR |
| hsa-miR-8057 | 3UTR |
| hsa-miR-8058 | 3UTR |
| hsa-miR-8059 | 3UTR |
| hsa-miR-8060 | 3UTR |
| hsa-miR-8069 | 3UTR |
| hsa-miR-8072 | 3UTR |
| hsa-miR-8073 | 3UTR |
| hsa-miR-8078 | 3UTR |
| hsa-miR-8081 | 3UTR |
| hsa-miR-8083 | 3UTR |
| hsa-miR-8085 | 3UTR |
| hsa-miR-9718 | 3UTR |
| hsa-miR-9898 | 3UTR |
| hsa-miR-9901 | 3UTR |
| hsa-miR-9903 | 3UTR |
| hsa-miR-1843 | 3UTR |
| hsa-miR-10392-3p | 3UTR |
| hsa-miR-10394-5p | 3UTR |
| hsa-miR-10395-5p | 3UTR |
| hsa-miR-10397-3p | 3UTR |
| hsa-miR-10398-5p | 3UTR |
| hsa-miR-10401-5p | 3UTR |
| hsa-miR-10401-3p | 3UTR |
| hsa-miR-10526-3p | 3UTR |
| hsa-miR-11181-5p | 3UTR |
| hsa-miR-11181-3p | 3UTR |
| hsa-miR-3059-3p | 3UTR |
| hsa-miR-3085-3p | 3UTR |
| hsa-miR-6529-5p | 3UTR |
| hsa-miR-9851-5p | 3UTR |
| hsa-miR-12114 | 3UTR |
| hsa-miR-12115 | 3UTR |
| hsa-miR-12116 | 3UTR |
| hsa-miR-12118 | 3UTR |
| hsa-miR-12119 | 3UTR |
| hsa-miR-12124 | 3UTR |
| hsa-miR-12128 | 3UTR |
| hsa-miR-12131 | 3UTR |
| hsa-miR-18a-5p | 3UTR |
| hsa-miR-24-1-5p | 3UTR |
| hsa-miR-98-5p | 3UTR |
| hsa-miR-210-5p | 3UTR |
| hsa-miR-215-3p | 3UTR |
| hsa-let-7g-3p | 3UTR |
| hsa-miR-141-5p | 3UTR |
| hsa-miR-143-5p | 3UTR |
| hsa-miR-143-3p | 3UTR |
| hsa-miR-136-3p | 3UTR |
| hsa-miR-149-5p | 3UTR |
| hsa-miR-185-5p | 3UTR |
| hsa-miR-200a-5p | 3UTR |
| hsa-miR-34c-5p | 3UTR |
| hsa-miR-301a-3p | 3UTR |
| hsa-miR-372-3p | 3UTR |
| hsa-miR-324-5p | 3UTR |
| hsa-miR-486-5p | 3UTR |
| hsa-miR-488-3p | 3UTR |
| hsa-miR-517-5p | 3UTR |
| hsa-miR-501-5p | 3UTR |
| hsa-miR-92b-3p | 3UTR |
| hsa-miR-616-3p | 3UTR |
| hsa-miR-647 | 3UTR |
| hsa-miR-449b-5p | 3UTR |
| hsa-miR-658 | 3UTR |
| hsa-miR-659-3p | 3UTR |
| hsa-miR-1298-3p | 3UTR |
| hsa-miR-874-3p | 3UTR |
| hsa-miR-147b-3p | 3UTR |
| hsa-miR-887-5p | 3UTR |
| hsa-miR-1228-3p | 3UTR |
| hsa-miR-1200 | 3UTR |
| hsa-miR-1268a | 3UTR |
| hsa-miR-1269a | 3UTR |
| hsa-miR-1270 | 3UTR |
| hsa-miR-1272 | 3UTR |
| hsa-miR-664a-3p | 3UTR |
| hsa-miR-2278 | 3UTR |
| hsa-miR-3125 | 3UTR |
| hsa-miR-3155a | 3UTR |
| hsa-miR-548w | 3UTR |
| hsa-miR-4302 | 3UTR |
| hsa-miR-4312 | 3UTR |
| hsa-miR-4251 | 3UTR |
| hsa-miR-3916 | 3UTR |
| hsa-miR-3922-3p | 3UTR |
| hsa-miR-4495 | 3UTR |
| hsa-miR-4517 | 3UTR |
| hsa-miR-4519 | 3UTR |
| hsa-miR-4677-5p | 3UTR |
| hsa-miR-4684-3p | 3UTR |
| hsa-miR-4700-3p | 3UTR |
| hsa-miR-4730 | 3UTR |
| hsa-miR-4742-5p | 3UTR |
| hsa-miR-4752 | 3UTR |
| hsa-miR-4768-5p | 3UTR |
| hsa-miR-4797-3p | 3UTR |
| hsa-miR-4799-3p | 3UTR |
| hsa-miR-5585-3p | 3UTR |
| hsa-miR-5587-3p | 3UTR |
| hsa-miR-5694 | 3UTR |
| hsa-miR-6070 | 3UTR |
| hsa-miR-6741-5p | 3UTR |
| hsa-miR-6771-3p | 3UTR |
| hsa-miR-6772-5p | 3UTR |
| hsa-miR-6772-3p | 3UTR |
| hsa-miR-6775-3p | 3UTR |
| hsa-miR-6784-5p | 3UTR |
| hsa-miR-6828-3p | 3UTR |
| hsa-miR-6835-5p | 3UTR |
| hsa-miR-6780b-5p | 3UTR |
| hsa-miR-6867-3p | 3UTR |
| hsa-miR-6871-3p | 3UTR |
| hsa-miR-6881-3p | 3UTR |
| hsa-miR-6883-3p | 3UTR |
| hsa-miR-6890-3p | 3UTR |
| hsa-miR-6894-3p | 3UTR |
| hsa-miR-7114-5p | 3UTR |
| hsa-miR-7154-5p | 3UTR |
| hsa-miR-7159-5p | 3UTR |
| hsa-miR-4433b-5p | 3UTR |
| hsa-miR-7854-3p | 3UTR |
| hsa-miR-7856-5p | 3UTR |
| hsa-miR-8052 | 3UTR |
| hsa-miR-8062 | 3UTR |
| hsa-miR-8075 | 3UTR |
| hsa-miR-11400 | 3UTR |
| hsa-miR-93-3p | 3UTR |
| hsa-miR-218-2-3p | 3UTR |
| hsa-miR-191-5p | 3UTR |
| hsa-miR-125a-3p | 3UTR |
| hsa-miR-30c-1-3p | 3UTR |
| hsa-miR-296-3p | 3UTR |
| hsa-miR-375-3p | 3UTR |
| hsa-miR-379-5p | 3UTR |
| hsa-miR-382-5p | 3UTR |
| hsa-miR-331-5p | 3UTR |
| hsa-miR-423-3p | 3UTR |
| hsa-miR-18b-5p | 3UTR |
| hsa-miR-489-5p | 3UTR |
| hsa-miR-493-5p | 3UTR |
| hsa-miR-521 | 3UTR |
| hsa-miR-505-5p | 3UTR |
| hsa-miR-550a-5p | 3UTR |
| hsa-miR-550a-3p | 3UTR |
| hsa-miR-600 | 3UTR |
| hsa-miR-621 | 3UTR |
| hsa-miR-622 | 3UTR |
| hsa-miR-636 | 3UTR |
| hsa-miR-758-5p | 3UTR |
| hsa-miR-449c-3p | 3UTR |
| hsa-miR-891b | 3UTR |
| hsa-miR-1251-3p | 3UTR |
| hsa-miR-1261 | 3UTR |
| hsa-miR-513c-5p | 3UTR |
| hsa-miR-1471 | 3UTR |
| hsa-miR-1908-3p | 3UTR |
| hsa-miR-1911-3p | 3UTR |
| hsa-miR-1913 | 3UTR |
| hsa-miR-1914-5p | 3UTR |
| hsa-miR-3158-5p | 3UTR |
| hsa-miR-3160-5p | 3UTR |
| hsa-miR-3180-3p | 3UTR |
| hsa-miR-3194-3p | 3UTR |
| hsa-miR-3198 | 3UTR |
| hsa-miR-4252 | 3UTR |
| hsa-miR-4326 | 3UTR |
| hsa-miR-4265 | 3UTR |
| hsa-miR-4268 | 3UTR |
| hsa-miR-4330 | 3UTR |
| hsa-miR-3661 | 3UTR |
| hsa-miR-3909 | 3UTR |
| hsa-miR-3922-5p | 3UTR |
| hsa-miR-676-5p | 3UTR |
| hsa-miR-3936 | 3UTR |
| hsa-miR-3944-5p | 3UTR |
| hsa-miR-4422 | 3UTR |
| hsa-miR-4447 | 3UTR |
| hsa-miR-4456 | 3UTR |
| hsa-miR-4465 | 3UTR |
| hsa-miR-4521 | 3UTR |
| hsa-miR-4527 | 3UTR |
| hsa-miR-4530 | 3UTR |
| hsa-miR-3972 | 3UTR |
| hsa-miR-4652-3p | 3UTR |
| hsa-miR-4653-3p | 3UTR |
| hsa-miR-4664-3p | 3UTR |
| hsa-miR-4668-5p | 3UTR |
| hsa-miR-4669 | 3UTR |
| hsa-miR-4707-3p | 3UTR |
| hsa-miR-4724-5p | 3UTR |
| hsa-miR-4727-5p | 3UTR |
| hsa-miR-4747-5p | 3UTR |
| hsa-miR-4755-5p | 3UTR |
| hsa-miR-4759 | 3UTR |
| hsa-miR-4769-3p | 3UTR |
| hsa-miR-4795-5p | 3UTR |
| hsa-miR-4797-5p | 3UTR |
| hsa-miR-5006-3p | 3UTR |
| hsa-miR-5588-5p | 3UTR |
| hsa-miR-5681a | 3UTR |
| hsa-miR-5693 | 3UTR |
| hsa-miR-5703 | 3UTR |
| hsa-miR-6073 | 3UTR |
| hsa-miR-6500-5p | 3UTR |
| hsa-miR-6716-5p | 3UTR |
| hsa-miR-6757-5p | 3UTR |
| hsa-miR-6765-5p | 3UTR |
| hsa-miR-6786-3p | 3UTR |
| hsa-miR-6796-5p | 3UTR |
| hsa-miR-6805-3p | 3UTR |
| hsa-miR-6833-3p | 3UTR |
| hsa-miR-6849-3p | 3UTR |
| hsa-miR-6854-3p | 3UTR |
| hsa-miR-6877-3p | 3UTR |
| hsa-miR-6879-3p | 3UTR |
| hsa-miR-6892-5p | 3UTR |
| hsa-miR-7109-3p | 3UTR |
| hsa-miR-7158-5p | 3UTR |
| hsa-miR-7706 | 3UTR |
| hsa-miR-6516-3p | 3UTR |
| hsa-miR-7976 | 3UTR |
| hsa-miR-9500 | 3UTR |
| hsa-miR-12122 | 3UTR |
| hsa-let-7e-5p | 3UTR |
| hsa-miR-16-1-3p | 3UTR |
| hsa-miR-33a-3p | 3UTR |
| hsa-miR-92a-1-5p | 3UTR |
| hsa-miR-198 | 3UTR |
| hsa-miR-200b-3p | 3UTR |
| hsa-miR-128-1-5p | 3UTR |
| hsa-miR-299-3p | 3UTR |
| hsa-miR-345-5p | 3UTR |
| hsa-miR-196b-5p | 3UTR |
| hsa-miR-422a | 3UTR |
| hsa-miR-423-5p | 3UTR |
| hsa-miR-432-3p | 3UTR |
| hsa-miR-502-5p | 3UTR |
| hsa-miR-376a-2-5p | 3UTR |
| hsa-miR-557 | 3UTR |
| hsa-miR-573 | 3UTR |
| hsa-miR-615-5p | 3UTR |
| hsa-miR-617 | 3UTR |
| hsa-miR-629-3p | 3UTR |
| hsa-miR-642a-3p | 3UTR |
| hsa-miR-663a | 3UTR |
| hsa-miR-767-3p | 3UTR |
| hsa-miR-764 | 3UTR |
| hsa-miR-759 | 3UTR |
| hsa-miR-874-5p | 3UTR |
| hsa-miR-744-3p | 3UTR |
| hsa-miR-933 | 3UTR |
| hsa-miR-939-5p | 3UTR |
| hsa-miR-939-3p | 3UTR |
| hsa-miR-940 | 3UTR |
| hsa-miR-1180-5p | 3UTR |
| hsa-miR-1225-3p | 3UTR |
| hsa-miR-1226-3p | 3UTR |
| hsa-miR-1229-5p | 3UTR |
| hsa-miR-1231 | 3UTR |
| hsa-miR-1276 | 3UTR |
| hsa-miR-1277-3p | 3UTR |
| hsa-miR-1538 | 3UTR |
| hsa-miR-1909-3p | 3UTR |
| hsa-miR-2682-5p | 3UTR |
| hsa-miR-3138 | 3UTR |
| hsa-miR-3151-5p | 3UTR |
| hsa-miR-3163 | 3UTR |
| hsa-miR-3165 | 3UTR |
| hsa-miR-1193 | 3UTR |
| hsa-miR-323b-5p | 3UTR |
| hsa-miR-323b-3p | 3UTR |
| hsa-miR-3175 | 3UTR |
| hsa-miR-3177-3p | 3UTR |
| hsa-miR-3187-3p | 3UTR |
| hsa-miR-3189-5p | 3UTR |
| hsa-miR-3191-3p | 3UTR |
| hsa-miR-4259 | 3UTR |
| hsa-miR-4253 | 3UTR |
| hsa-miR-2355-5p | 3UTR |
| hsa-miR-4274 | 3UTR |
| hsa-miR-4284 | 3UTR |
| hsa-miR-4292 | 3UTR |
| hsa-miR-3610 | 3UTR |
| hsa-miR-3620-5p | 3UTR |
| hsa-miR-3654 | 3UTR |
| hsa-miR-3682-3p | 3UTR |
| hsa-miR-3688-3p | 3UTR |
| hsa-miR-3907 | 3UTR |
| hsa-miR-3923 | 3UTR |
| hsa-miR-642b-3p | 3UTR |
| hsa-miR-4444 | 3UTR |
| hsa-miR-378h | 3UTR |
| hsa-miR-4466 | 3UTR |
| hsa-miR-4492 | 3UTR |
| hsa-miR-4497 | 3UTR |
| hsa-miR-4505 | 3UTR |
| hsa-miR-4507 | 3UTR |
| hsa-miR-4508 | 3UTR |
| hsa-miR-4514 | 3UTR |
| hsa-miR-4535 | 3UTR |
| hsa-miR-1587 | 3UTR |
| hsa-miR-4536-5p | 3UTR |
| hsa-miR-4642 | 3UTR |
| hsa-miR-4647 | 3UTR |
| hsa-miR-4651 | 3UTR |
| hsa-miR-4665-5p | 3UTR |
| hsa-miR-4683 | 3UTR |
| hsa-miR-1343-5p | 3UTR |
| hsa-miR-4707-5p | 3UTR |
| hsa-miR-4713-5p | 3UTR |
| hsa-miR-4717-3p | 3UTR |
| hsa-miR-4726-3p | 3UTR |
| hsa-miR-4746-5p | 3UTR |
| hsa-miR-4746-3p | 3UTR |
| hsa-miR-4750-5p | 3UTR |
| hsa-miR-4754 | 3UTR |
| hsa-miR-4763-5p | 3UTR |
| hsa-miR-4783-5p | 3UTR |
| hsa-miR-4783-3p | 3UTR |
| hsa-miR-4790-3p | 3UTR |
| hsa-miR-4793-5p | 3UTR |
| hsa-miR-4796-3p | 3UTR |
| hsa-miR-5000-3p | 3UTR |
| hsa-miR-5002-5p | 3UTR |
| hsa-miR-5008-3p | 3UTR |
| hsa-miR-5011-3p | 3UTR |
| hsa-miR-5189-5p | 3UTR |
| hsa-miR-1295b-5p | 3UTR |
| hsa-miR-5588-3p | 3UTR |
| hsa-miR-5696 | 3UTR |
| hsa-miR-5698 | 3UTR |
| hsa-miR-5706 | 3UTR |
| hsa-miR-6077 | 3UTR |
| hsa-miR-6125 | 3UTR |
| hsa-miR-6132 | 3UTR |
| hsa-miR-6514-3p | 3UTR |
| hsa-miR-6722-5p | 3UTR |
| hsa-miR-6735-5p | 3UTR |
| hsa-miR-6741-3p | 3UTR |
| hsa-miR-6759-5p | 3UTR |
| hsa-miR-6764-5p | 3UTR |
| hsa-miR-6765-3p | 3UTR |
| hsa-miR-6766-5p | 3UTR |
| hsa-miR-6802-5p | 3UTR |
| hsa-miR-6803-5p | 3UTR |
| hsa-miR-6820-5p | 3UTR |
| hsa-miR-6823-5p | 3UTR |
| hsa-miR-6842-3p | 3UTR |
| hsa-miR-6871-5p | 3UTR |
| hsa-miR-6893-5p | 3UTR |
| hsa-miR-7974 | 3UTR |
| hsa-miR-8071 | 3UTR |
| hsa-miR-8086 | 3UTR |
| hsa-miR-8088 | 3UTR |
| hsa-miR-10226 | 3UTR |
| hsa-miR-10400-3p | 3UTR |
| hsa-miR-10396b-3p | 3UTR |
| hsa-miR-11399 | 3UTR |
| hsa-miR-12129 | 3UTR |
| hsa-miR-12136 | 3UTR |
| hsa-let-7c-3p | 3UTR |
| hsa-let-7d-3p | 3UTR |
| hsa-let-7f-2-3p | 3UTR |
| hsa-miR-17-5p | 3UTR |
| hsa-miR-17-3p | 3UTR |
| hsa-miR-20a-3p | 3UTR |
| hsa-miR-22-5p | 3UTR |
| hsa-miR-22-3p | 3UTR |
| hsa-miR-23a-5p | 3UTR |
| hsa-miR-27a-3p | 3UTR |
| hsa-miR-28-5p | 3UTR |
| hsa-miR-32-3p | 3UTR |
| hsa-miR-33a-5p | 3UTR |
| hsa-miR-92a-3p | 3UTR |
| hsa-miR-96-5p | 3UTR |
| hsa-miR-99a-5p | 3UTR |
| hsa-miR-101-5p | 3UTR |
| hsa-miR-101-3p | 3UTR |
| hsa-miR-29b-2-5p | 3UTR |
| hsa-miR-105-5p | 3UTR |
| hsa-miR-105-3p | 3UTR |
| hsa-miR-106a-5p | 3UTR |
| hsa-miR-197-5p | 3UTR |
| hsa-miR-208a-5p | 3UTR |
| hsa-miR-129-5p | 3UTR |
| hsa-miR-129-1-3p | 3UTR |
| hsa-miR-148a-5p | 3UTR |
| hsa-miR-148a-3p | 3UTR |
| hsa-miR-7-2-3p | 3UTR |
| hsa-miR-10a-5p | 3UTR |
| hsa-miR-10b-3p | 3UTR |
| hsa-miR-34a-5p | 3UTR |
| hsa-miR-34a-3p | 3UTR |
| hsa-miR-181a-2-3p | 3UTR |
| hsa-miR-181b-5p | 3UTR |
| hsa-miR-181c-5p | 3UTR |
| hsa-miR-212-3p | 3UTR |
| hsa-miR-216a-5p | 3UTR |
| hsa-miR-217-5p | 3UTR |
| hsa-miR-218-1-3p | 3UTR |
| hsa-miR-223-3p | 3UTR |
| hsa-miR-224-5p | 3UTR |
| hsa-miR-15b-3p | 3UTR |
| hsa-miR-27b-3p | 3UTR |
| hsa-miR-122-5p | 3UTR |
| hsa-miR-125b-5p | 3UTR |
| hsa-miR-130a-3p | 3UTR |
| hsa-miR-141-3p | 3UTR |
| hsa-miR-152-5p | 3UTR |
| hsa-miR-153-5p | 3UTR |
| hsa-miR-9-5p | 3UTR |
| hsa-miR-125a-5p | 3UTR |
| hsa-miR-129-2-3p | 3UTR |
| hsa-miR-138-1-3p | 3UTR |
| hsa-miR-146a-3p | 3UTR |
| hsa-miR-149-3p | 3UTR |
| hsa-miR-154-5p | 3UTR |
| hsa-miR-184 | 3UTR |
| hsa-miR-188-5p | 3UTR |
| hsa-miR-193a-3p | 3UTR |
| hsa-miR-195-5p | 3UTR |
| hsa-miR-195-3p | 3UTR |
| hsa-miR-1-5p | 3UTR |
| hsa-miR-181b-2-3p | 3UTR |
| hsa-miR-29c-3p | 3UTR |
| hsa-miR-200a-3p | 3UTR |
| hsa-miR-101-2-5p | 3UTR |
| hsa-miR-34b-3p | 3UTR |
| hsa-miR-301a-5p | 3UTR |
| hsa-miR-130b-5p | 3UTR |
| hsa-miR-30e-5p | 3UTR |
| hsa-miR-26a-2-3p | 3UTR |
| hsa-miR-361-3p | 3UTR |
| hsa-miR-362-5p | 3UTR |
| hsa-miR-363-3p | 3UTR |
| hsa-miR-302d-3p | 3UTR |
| hsa-miR-367-5p | 3UTR |
| hsa-miR-370-5p | 3UTR |
| hsa-miR-373-5p | 3UTR |
| hsa-miR-375-5p | 3UTR |
| hsa-miR-378a-3p | 3UTR |
| hsa-miR-379-3p | 3UTR |
| hsa-miR-380-5p | 3UTR |
| hsa-miR-383-3p | 3UTR |
| hsa-miR-340-3p | 3UTR |
| hsa-miR-328-5p | 3UTR |
| hsa-miR-328-3p | 3UTR |
| hsa-miR-342-3p | 3UTR |
| hsa-miR-151a-5p | 3UTR |
| hsa-miR-338-3p | 3UTR |
| hsa-miR-133b | 3UTR |
| hsa-miR-20b-5p | 3UTR |
| hsa-miR-449a | 3UTR |
| hsa-miR-329-3p | 3UTR |
| hsa-miR-452-5p | 3UTR |
| hsa-miR-409-5p | 3UTR |
| hsa-miR-412-3p | 3UTR |
| hsa-miR-483-3p | 3UTR |
| hsa-miR-486-3p | 3UTR |
| hsa-miR-487a-5p | 3UTR |
| hsa-miR-488-5p | 3UTR |
| hsa-miR-491-3p | 3UTR |
| hsa-miR-495-5p | 3UTR |
| hsa-miR-193b-3p | 3UTR |
| hsa-miR-512-5p | 3UTR |
| hsa-miR-512-3p | 3UTR |
| hsa-miR-498-5p | 3UTR |
| hsa-miR-519c-5p | 3UTR |
| hsa-miR-519c-3p | 3UTR |
| hsa-miR-526b-5p | 3UTR |
| hsa-miR-519b-5p | 3UTR |
| hsa-miR-523-5p | 3UTR |
| hsa-miR-523-3p | 3UTR |
| hsa-miR-518f-5p | 3UTR |
| hsa-miR-518f-3p | 3UTR |
| hsa-miR-520b-5p | 3UTR |
| hsa-miR-526a-5p | 3UTR |
| hsa-miR-520c-5p | 3UTR |
| hsa-miR-518c-5p | 3UTR |
| hsa-miR-520d-5p | 3UTR |
| hsa-miR-516b-5p | 3UTR |
| hsa-miR-518e-5p | 3UTR |
| hsa-miR-518a-5p | 3UTR |
| hsa-miR-518d-5p | 3UTR |
| hsa-miR-518d-3p | 3UTR |
| hsa-miR-517c-3p | 3UTR |
| hsa-miR-522-5p | 3UTR |
| hsa-miR-519a-5p | 3UTR |
| hsa-miR-519a-3p | 3UTR |
| hsa-miR-527 | 3UTR |
| hsa-miR-519a-2-5p | 3UTR |
| hsa-miR-499a-3p | 3UTR |
| hsa-miR-500a-3p | 3UTR |
| hsa-miR-502-3p | 3UTR |
| hsa-miR-450a-2-3p | 3UTR |
| hsa-miR-504-5p | 3UTR |
| hsa-miR-506-5p | 3UTR |
| hsa-miR-509-5p | 3UTR |
| hsa-miR-510-5p | 3UTR |
| hsa-miR-510-3p | 3UTR |
| hsa-miR-514a-5p | 3UTR |
| hsa-miR-514a-3p | 3UTR |
| hsa-miR-487b-5p | 3UTR |
| hsa-miR-551a | 3UTR |
| hsa-miR-552-5p | 3UTR |
| hsa-miR-555 | 3UTR |
| hsa-miR-562 | 3UTR |
| hsa-miR-567 | 3UTR |
| hsa-miR-569 | 3UTR |
| hsa-miR-571 | 3UTR |
| hsa-miR-572 | 3UTR |
| hsa-miR-574-3p | 3UTR |
| hsa-miR-578 | 3UTR |
| hsa-miR-584-5p | 3UTR |
| hsa-miR-588 | 3UTR |
| hsa-miR-589-3p | 3UTR |
| hsa-miR-593-5p | 3UTR |
| hsa-miR-598-3p | 3UTR |
| hsa-miR-601 | 3UTR |
| hsa-miR-605-3p | 3UTR |
| hsa-miR-608 | 3UTR |
| hsa-miR-611 | 3UTR |
| hsa-miR-612 | 3UTR |
| hsa-miR-613 | 3UTR |
| hsa-miR-614 | 3UTR |
| hsa-miR-618 | 3UTR |
| hsa-miR-619-3p | 3UTR |
| hsa-miR-624-5p | 3UTR |
| hsa-miR-624-3p | 3UTR |
| hsa-miR-627-3p | 3UTR |
| hsa-miR-33b-5p | 3UTR |
| hsa-miR-644a | 3UTR |
| hsa-miR-645 | 3UTR |
| hsa-miR-652-3p | 3UTR |
| hsa-miR-661 | 3UTR |
| hsa-miR-411-3p | 3UTR |
| hsa-miR-655-5p | 3UTR |
| hsa-miR-542-5p | 3UTR |
| hsa-miR-542-3p | 3UTR |
| hsa-miR-550a-3-5p | 3UTR |
| hsa-miR-151b | 3UTR |
| hsa-miR-1271-5p | 3UTR |
| hsa-miR-454-3p | 3UTR |
| hsa-miR-769-3p | 3UTR |
| hsa-miR-762 | 3UTR |
| hsa-miR-670-3p | 3UTR |
| hsa-miR-675-3p | 3UTR |
| hsa-miR-890 | 3UTR |
| hsa-miR-889-3p | 3UTR |
| hsa-miR-708-5p | 3UTR |
| hsa-miR-885-5p | 3UTR |
| hsa-miR-208b-5p | 3UTR |
| hsa-miR-924 | 3UTR |
| hsa-miR-934 | 3UTR |
| hsa-miR-942-5p | 3UTR |
| hsa-miR-943 | 3UTR |
| hsa-miR-1178-3p | 3UTR |
| hsa-miR-1179 | 3UTR |
| hsa-miR-1182 | 3UTR |
| hsa-miR-1184 | 3UTR |
| hsa-miR-1234-3p | 3UTR |
| hsa-miR-1237-3p | 3UTR |
| hsa-miR-1208 | 3UTR |
| hsa-miR-548e-5p | 3UTR |
| hsa-miR-548e-3p | 3UTR |
| hsa-miR-1291 | 3UTR |
| hsa-miR-548k | 3UTR |
| hsa-miR-1294 | 3UTR |
| hsa-miR-1305 | 3UTR |
| hsa-miR-1247-5p | 3UTR |
| hsa-miR-1248 | 3UTR |
| hsa-miR-1249-5p | 3UTR |
| hsa-miR-1255a | 3UTR |
| hsa-miR-548o-3p | 3UTR |
| hsa-miR-1266-3p | 3UTR |
| hsa-miR-1282 | 3UTR |
| hsa-miR-1292-3p | 3UTR |
| hsa-miR-1306-3p | 3UTR |
| hsa-miR-1307-3p | 3UTR |
| hsa-miR-1322 | 3UTR |
| hsa-miR-1324 | 3UTR |
| hsa-miR-1537-5p | 3UTR |
| hsa-miR-1539 | 3UTR |
| hsa-miR-103b | 3UTR |
| hsa-miR-1914-3p | 3UTR |
| hsa-miR-2115-5p | 3UTR |
| hsa-miR-2115-3p | 3UTR |
| hsa-miR-2116-3p | 3UTR |
| hsa-miR-2277-3p | 3UTR |
| hsa-miR-2681-3p | 3UTR |
| hsa-miR-711 | 3UTR |
| hsa-miR-3116 | 3UTR |
| hsa-miR-3121-5p | 3UTR |
| hsa-miR-3124-5p | 3UTR |
| hsa-miR-3124-3p | 3UTR |
| hsa-miR-548s | 3UTR |
| hsa-miR-3126-5p | 3UTR |
| hsa-miR-3126-3p | 3UTR |
| hsa-miR-3127-5p | 3UTR |
| hsa-miR-3127-3p | 3UTR |
| hsa-miR-3136-5p | 3UTR |
| hsa-miR-3136-3p | 3UTR |
| hsa-miR-3141 | 3UTR |
| hsa-miR-3143 | 3UTR |
| hsa-miR-1273c | 3UTR |
| hsa-miR-3151-3p | 3UTR |
| hsa-miR-3152-5p | 3UTR |
| hsa-miR-3074-3p | 3UTR |
| hsa-miR-3159 | 3UTR |
| hsa-miR-3166 | 3UTR |
| hsa-miR-3169 | 3UTR |
| hsa-miR-3176 | 3UTR |
| hsa-miR-3184-5p | 3UTR |
| hsa-miR-3184-3p | 3UTR |
| hsa-miR-3186-5p | 3UTR |
| hsa-miR-3187-5p | 3UTR |
| hsa-miR-3189-3p | 3UTR |
| hsa-miR-3191-5p | 3UTR |
| hsa-miR-3192-3p | 3UTR |
| hsa-miR-3194-5p | 3UTR |
| hsa-miR-3195 | 3UTR |
| hsa-miR-3200-3p | 3UTR |
| hsa-miR-4296 | 3UTR |
| hsa-miR-378c | 3UTR |
| hsa-miR-4293 | 3UTR |
| hsa-miR-4304 | 3UTR |
| hsa-miR-4303 | 3UTR |
| hsa-miR-4305 | 3UTR |
| hsa-miR-4307 | 3UTR |
| hsa-miR-4308 | 3UTR |
| hsa-miR-4311 | 3UTR |
| hsa-miR-4313 | 3UTR |
| hsa-miR-4316 | 3UTR |
| hsa-miR-4314 | 3UTR |
| hsa-miR-4319 | 3UTR |
| hsa-miR-4320 | 3UTR |
| hsa-miR-4256 | 3UTR |
| hsa-miR-4260 | 3UTR |
| hsa-miR-4261 | 3UTR |
| hsa-miR-4266 | 3UTR |
| hsa-miR-2355-3p | 3UTR |
| hsa-miR-4269 | 3UTR |
| hsa-miR-4263 | 3UTR |
| hsa-miR-4273 | 3UTR |
| hsa-miR-4278 | 3UTR |
| hsa-miR-4285 | 3UTR |
| hsa-miR-4283 | 3UTR |
| hsa-miR-4286 | 3UTR |
| hsa-miR-4288 | 3UTR |
| hsa-miR-4329 | 3UTR |
| hsa-miR-500b-3p | 3UTR |
| hsa-miR-3615 | 3UTR |
| hsa-miR-23c | 3UTR |
| hsa-miR-3620-3p | 3UTR |
| hsa-miR-3622a-3p | 3UTR |
| hsa-miR-3622b-5p | 3UTR |
| hsa-miR-3622b-3p | 3UTR |
| hsa-miR-3649 | 3UTR |
| hsa-miR-3662 | 3UTR |
| hsa-miR-3664-5p | 3UTR |
| hsa-miR-3675-3p | 3UTR |
| hsa-miR-3677-5p | 3UTR |
| hsa-miR-3679-5p | 3UTR |
| hsa-miR-3921 | 3UTR |
| hsa-miR-3924 | 3UTR |
| hsa-miR-3927-5p | 3UTR |
| hsa-miR-3929 | 3UTR |
| hsa-miR-550b-3p | 3UTR |
| hsa-miR-4418 | 3UTR |
| hsa-miR-4420 | 3UTR |
| hsa-miR-4426 | 3UTR |
| hsa-miR-4432 | 3UTR |
| hsa-miR-4433a-3p | 3UTR |
| hsa-miR-4440 | 3UTR |
| hsa-miR-4443 | 3UTR |
| hsa-miR-548ah-5p | 3UTR |
| hsa-miR-4452 | 3UTR |
| hsa-miR-4457 | 3UTR |
| hsa-miR-4460 | 3UTR |
| hsa-miR-4469 | 3UTR |
| hsa-miR-4474-3p | 3UTR |
| hsa-miR-3689f | 3UTR |
| hsa-miR-4481 | 3UTR |
| hsa-miR-4486 | 3UTR |
| hsa-miR-4501 | 3UTR |
| hsa-miR-4502 | 3UTR |
| hsa-miR-4512 | 3UTR |
| hsa-miR-4515 | 3UTR |
| hsa-miR-4522 | 3UTR |
| hsa-miR-4533 | 3UTR |
| hsa-miR-378i | 3UTR |
| hsa-miR-548am-3p | 3UTR |
| hsa-miR-548an | 3UTR |
| hsa-miR-4539 | 3UTR |
| hsa-miR-4540 | 3UTR |
| hsa-miR-3973 | 3UTR |
| hsa-miR-4633-5p | 3UTR |
| hsa-miR-4634 | 3UTR |
| hsa-miR-4639-3p | 3UTR |
| hsa-miR-4641 | 3UTR |
| hsa-miR-4648 | 3UTR |
| hsa-miR-4652-5p | 3UTR |
| hsa-miR-4655-3p | 3UTR |
| hsa-miR-4659b-3p | 3UTR |
| hsa-miR-4667-5p | 3UTR |
| hsa-miR-4667-3p | 3UTR |
| hsa-miR-4668-3p | 3UTR |
| hsa-miR-4680-5p | 3UTR |
| hsa-miR-4682 | 3UTR |
| hsa-miR-1343-3p | 3UTR |
| hsa-miR-4691-5p | 3UTR |
| hsa-miR-4693-5p | 3UTR |
| hsa-miR-4696 | 3UTR |
| hsa-miR-4697-3p | 3UTR |
| hsa-miR-4699-3p | 3UTR |
| hsa-miR-203b-5p | 3UTR |
| hsa-miR-4710 | 3UTR |
| hsa-miR-4712-5p | 3UTR |
| hsa-miR-4713-3p | 3UTR |
| hsa-miR-4714-5p | 3UTR |
| hsa-miR-3529-5p | 3UTR |
| hsa-miR-4722-3p | 3UTR |
| hsa-miR-4723-3p | 3UTR |
| hsa-miR-4731-5p | 3UTR |
| hsa-miR-4735-5p | 3UTR |
| hsa-miR-4738-3p | 3UTR |
| hsa-miR-4740-5p | 3UTR |
| hsa-miR-4742-3p | 3UTR |
| hsa-miR-4743-3p | 3UTR |
| hsa-miR-4749-3p | 3UTR |
| hsa-miR-4751 | 3UTR |
| hsa-miR-4753-3p | 3UTR |
| hsa-miR-499b-3p | 3UTR |
| hsa-miR-4758-5p | 3UTR |
| hsa-miR-4758-3p | 3UTR |
| hsa-miR-4761-5p | 3UTR |
| hsa-miR-4762-3p | 3UTR |
| hsa-miR-4763-3p | 3UTR |
| hsa-miR-4764-3p | 3UTR |
| hsa-miR-4765 | 3UTR |
| hsa-miR-4766-5p | 3UTR |
| hsa-miR-4774-5p | 3UTR |
| hsa-miR-4780 | 3UTR |
| hsa-miR-4782-3p | 3UTR |
| hsa-miR-4789-5p | 3UTR |
| hsa-miR-4798-5p | 3UTR |
| hsa-miR-4804-3p | 3UTR |
| hsa-miR-4999-5p | 3UTR |
| hsa-miR-5000-5p | 3UTR |
| hsa-miR-5003-3p | 3UTR |
| hsa-miR-5004-3p | 3UTR |
| hsa-miR-548ao-5p | 3UTR |
| hsa-miR-5007-5p | 3UTR |
| hsa-miR-5088-5p | 3UTR |
| hsa-miR-5088-3p | 3UTR |
| hsa-miR-5092 | 3UTR |
| hsa-miR-5186 | 3UTR |
| hsa-miR-5187-3p | 3UTR |
| hsa-miR-5194 | 3UTR |
| hsa-miR-5195-5p | 3UTR |
| hsa-miR-5197-3p | 3UTR |
| hsa-miR-4524b-3p | 3UTR |
| hsa-miR-5571-3p | 3UTR |
| hsa-miR-5581-5p | 3UTR |
| hsa-miR-5581-3p | 3UTR |
| hsa-miR-548at-3p | 3UTR |
| hsa-miR-5582-3p | 3UTR |
| hsa-miR-5586-3p | 3UTR |
| hsa-miR-5587-5p | 3UTR |
| hsa-miR-5591-3p | 3UTR |
| hsa-miR-548av-3p | 3UTR |
| hsa-miR-5685 | 3UTR |
| hsa-miR-5687 | 3UTR |
| hsa-miR-5690 | 3UTR |
| hsa-miR-5701 | 3UTR |
| hsa-miR-5702 | 3UTR |
| hsa-miR-5787 | 3UTR |
| hsa-miR-1199-5p | 3UTR |
| hsa-miR-6069 | 3UTR |
| hsa-miR-6075 | 3UTR |
| hsa-miR-6081 | 3UTR |
| hsa-miR-6084 | 3UTR |
| hsa-miR-6085 | 3UTR |
| hsa-miR-6089 | 3UTR |
| hsa-miR-6126 | 3UTR |
| hsa-miR-6128 | 3UTR |
| hsa-miR-378j | 3UTR |
| hsa-miR-548az-5p | 3UTR |
| hsa-miR-6501-3p | 3UTR |
| hsa-miR-6504-5p | 3UTR |
| hsa-miR-6505-3p | 3UTR |
| hsa-miR-6506-5p | 3UTR |
| hsa-miR-6507-5p | 3UTR |
| hsa-miR-6511a-3p | 3UTR |
| hsa-miR-6512-5p | 3UTR |
| hsa-miR-6512-3p | 3UTR |
| hsa-miR-6513-3p | 3UTR |
| hsa-miR-6515-3p | 3UTR |
| hsa-miR-6715b-5p | 3UTR |
| hsa-miR-6716-3p | 3UTR |
| hsa-miR-6718-5p | 3UTR |
| hsa-miR-892c-5p | 3UTR |
| hsa-miR-892c-3p | 3UTR |
| hsa-miR-6726-3p | 3UTR |
| hsa-miR-6727-5p | 3UTR |
| hsa-miR-6727-3p | 3UTR |
| hsa-miR-6729-3p | 3UTR |
| hsa-miR-6730-3p | 3UTR |
| hsa-miR-6731-3p | 3UTR |
| hsa-miR-6736-5p | 3UTR |
| hsa-miR-6738-3p | 3UTR |
| hsa-miR-6743-5p | 3UTR |
| hsa-miR-6744-5p | 3UTR |
| hsa-miR-6749-3p | 3UTR |
| hsa-miR-6752-3p | 3UTR |
| hsa-miR-6753-3p | 3UTR |
| hsa-miR-6757-3p | 3UTR |
| hsa-miR-6760-3p | 3UTR |
| hsa-miR-6761-3p | 3UTR |
| hsa-miR-6763-5p | 3UTR |
| hsa-miR-6767-5p | 3UTR |
| hsa-miR-6768-5p | 3UTR |
| hsa-miR-6768-3p | 3UTR |
| hsa-miR-6769a-5p | 3UTR |
| hsa-miR-6777-3p | 3UTR |
| hsa-miR-6788-3p | 3UTR |
| hsa-miR-6792-3p | 3UTR |
| hsa-miR-6793-3p | 3UTR |
| hsa-miR-6794-3p | 3UTR |
| hsa-miR-6795-3p | 3UTR |
| hsa-miR-6796-3p | 3UTR |
| hsa-miR-6797-3p | 3UTR |
| hsa-miR-6801-3p | 3UTR |
| hsa-miR-6802-3p | 3UTR |
| hsa-miR-6806-5p | 3UTR |
| hsa-miR-6806-3p | 3UTR |
| hsa-miR-6809-5p | 3UTR |
| hsa-miR-6812-3p | 3UTR |
| hsa-miR-6813-3p | 3UTR |
| hsa-miR-6814-3p | 3UTR |
| hsa-miR-6817-3p | 3UTR |
| hsa-miR-6826-3p | 3UTR |
| hsa-miR-6827-3p | 3UTR |
| hsa-miR-6828-5p | 3UTR |
| hsa-miR-6832-3p | 3UTR |
| hsa-miR-6834-5p | 3UTR |
| hsa-miR-6834-3p | 3UTR |
| hsa-miR-6845-3p | 3UTR |
| hsa-miR-6847-3p | 3UTR |
| hsa-miR-6848-5p | 3UTR |
| hsa-miR-6851-3p | 3UTR |
| hsa-miR-6852-3p | 3UTR |
| hsa-miR-6856-3p | 3UTR |
| hsa-miR-6858-3p | 3UTR |
| hsa-miR-6859-3p | 3UTR |
| hsa-miR-6862-3p | 3UTR |
| hsa-miR-6867-5p | 3UTR |
| hsa-miR-6868-5p | 3UTR |
| hsa-miR-6868-3p | 3UTR |
| hsa-miR-6875-5p | 3UTR |
| hsa-miR-6877-5p | 3UTR |
| hsa-miR-6884-3p | 3UTR |
| hsa-miR-6885-3p | 3UTR |
| hsa-miR-6886-3p | 3UTR |
| hsa-miR-6887-3p | 3UTR |
| hsa-miR-6895-3p | 3UTR |
| hsa-miR-7107-3p | 3UTR |
| hsa-miR-7111-3p | 3UTR |
| hsa-miR-7151-5p | 3UTR |
| hsa-miR-7152-5p | 3UTR |
| hsa-miR-7152-3p | 3UTR |
| hsa-miR-7157-3p | 3UTR |
| hsa-miR-7161-5p | 3UTR |
| hsa-miR-7161-3p | 3UTR |
| hsa-miR-7702 | 3UTR |
| hsa-miR-7705 | 3UTR |
| hsa-miR-1273h-3p | 3UTR |
| hsa-miR-7851-3p | 3UTR |
| hsa-miR-7975 | 3UTR |
| hsa-miR-8056 | 3UTR |
| hsa-miR-8061 | 3UTR |
| hsa-miR-8063 | 3UTR |
| hsa-miR-8065 | 3UTR |
| hsa-miR-8074 | 3UTR |
| hsa-miR-8076 | 3UTR |
| hsa-miR-8077 | 3UTR |
| hsa-miR-8079 | 3UTR |
| hsa-miR-8082 | 3UTR |
| hsa-miR-8089 | 3UTR |
| hsa-miR-9985 | 3UTR |
| hsa-miR-9986 | 3UTR |
| hsa-miR-10392-5p | 3UTR |
| hsa-miR-10396a-3p | 3UTR |
| hsa-miR-10397-5p | 3UTR |
| hsa-miR-10398-3p | 3UTR |
| hsa-miR-10399-5p | 3UTR |
| hsa-miR-10399-3p | 3UTR |
| hsa-miR-10523-5p | 3UTR |
| hsa-miR-9983-3p | 3UTR |
| hsa-miR-11401 | 3UTR |
| hsa-miR-3059-5p | 3UTR |
| hsa-miR-3085-5p | 3UTR |
| hsa-miR-6529-3p | 3UTR |
| hsa-miR-12120 | 3UTR |
| hsa-miR-12121 | 3UTR |
| hsa-miR-12125 | 3UTR |
| hsa-miR-12126 | 3UTR |
| hsa-miR-12127 | 3UTR |
| hsa-miR-12133 | 3UTR |
