# Supplementary figures and images for: Long non-coding RNAs PGM5-AS1 upregulates Decorin (DCN) to inhibit cervical cancer progression by sponging miR-4284
Source: Bioengineered. 2022 Apr 14;13(4):9872–84. doi: 10.1080/21655979.2022.2062088 (PMC9161867; doi:10.1080/21655979.2022.2062088)

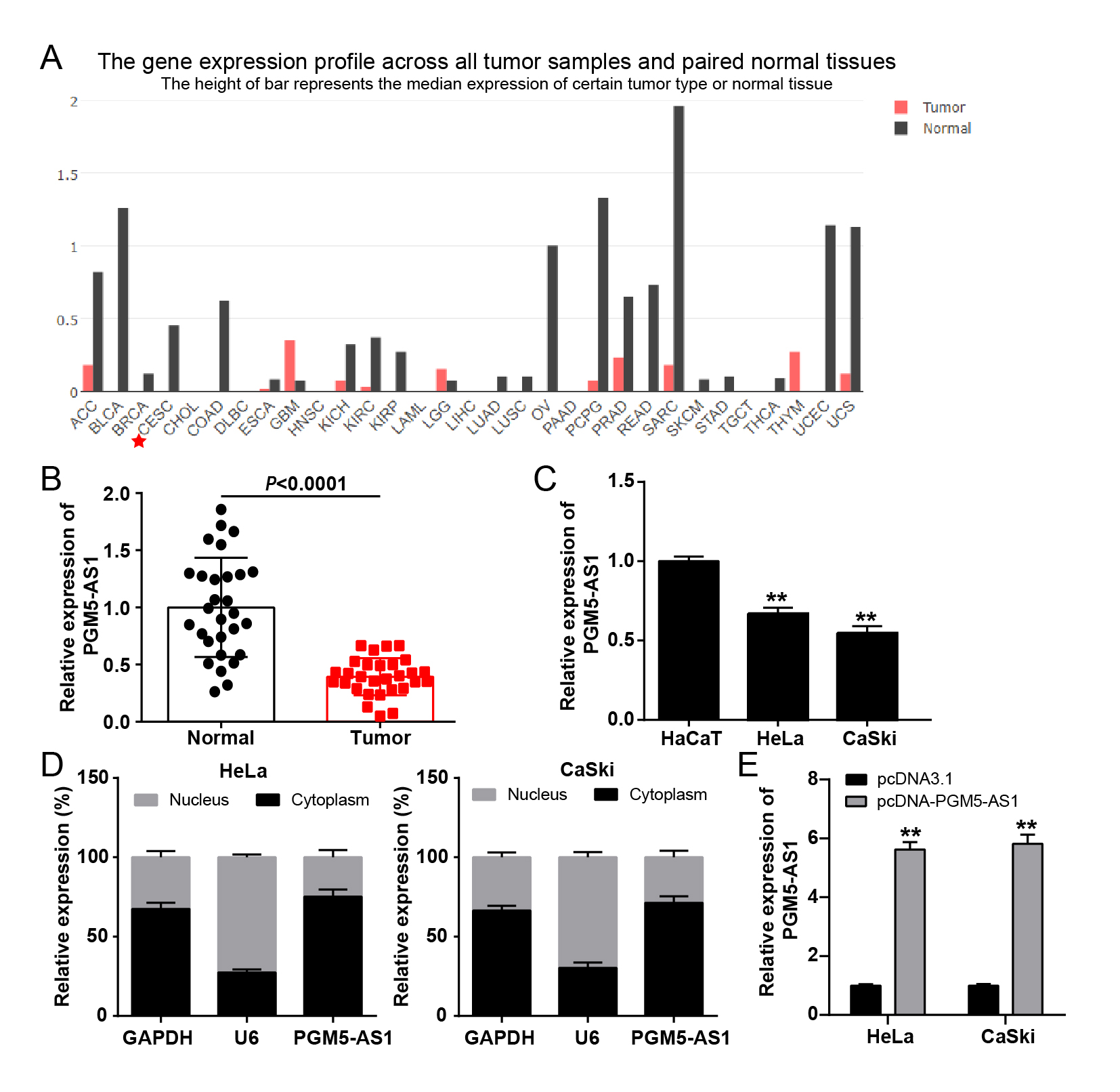

Supplement: Supplemental Material [file KBIE_A_2062088_SM1689.zip › supplementary/Supplementary Figure 1.jpg]
